# Mental health problems and suicidal behaviors in person-related work: a Swedish register-based cohort study

**DOI:** 10.5271/sjweh.4316

**Published:** 2026-09-01

**Authors:** Kuan-Yu Pan, Alicia Nevriana, Katrina Blindow, Melody Almroth, Katarina Kjellberg, Daniel Falkstedt

**Affiliations:** 1 Institute of Environmental Medicine, Karolinska Institutet, Stockholm, Sweden.; 2 German Youth Institute (DJI), Munich, Germany.; 3 The Centre for Occupational and Environmental Medicine, Region Stockholm, Stockholm, Sweden.

**Keywords:** alcohol use disorder, anxiety disorder, depression, drug use disorder, emotional demand, human contact occupation, stress-related disorder, suicide

## Abstract

**Objectives:**

Person-related work requires workers to interact with individuals who are not employed at the workplace, eg, clients or patients. Besides (i) the general demands of interpersonal contact, this can entail (ii) emotional demands and (iii) conflicts/quarrels. These three potential stressors may negatively affect workers’ mental health. We examined the associations of these three dimensions of person-related work with the risk of mental health problems, including diagnoses of depressive, anxiety/stress-related and alcohol-/drug-use disorders, pharmacotherapy for such disorders, and suicidal behaviors, including deaths and suicide attempts.

**Methods:**

Around 3.6 million workers aged 20–60 years in Sweden in 2006 were included in the study. Dimensions of person-related work were assessed respectively using a job exposure matrix. Mental health and suicide outcomes in 2007–2020 were determined based on patient, drug, and death registers. Multi-variable Cox regression models were used.

**Results:**

Of the study participants, 1 481 900 individuals experienced at least one of the studied outcomes. Among women, high exposure to each dimension of person-related work was respectively associated with increased risks of the outcomes [hazard ratios (HR) of 1.04–1.27 for general contact with people, 1.16–1.41 for emotional demands, and 1.11–1.19 for conflicts/quarrels], after adjusting for potential confounders. Among men, a high exposure to each dimension was respectively associated with increased risks (HR of 1.11–1.16 for general contact with people, 1.10–1.23 for emotional demands, and 1.08–1.16 for conflicts/quarrels) of mental health diagnoses and pharmacotherapy. The associations with suicide deaths and attempts were not observed after adjusting for income and previous mental health problems and suicide attempts.

**Conclusions:**

Person-related work is associated with a slightly increased risk of mental health problems and suicidal behaviors among women and with mental health problems among men. Estimates seem to be strongest for emotional demands.

Mental health problems and suicidal behaviors are major public health concerns that are gaining attention in workplace settings throughout the world (1, 2). The World Health Organization estimated that, in a company of 1000 employees, 200–300 workers will suffer from a serious mental health problem in any given year, 1 worker will die by suicide every ten years, and for every employee who dies by suicide, another 10–20 will attempt suicide (3).

While causes for mental health problems and suicidal behaviors are multifaceted, adverse psychosocial working conditions have been suggested to be among the contributing factors (4). A recent umbrella review, based on seven systematic reviews, summarized that job strain, effort–reward imbalance, low procedural justice, and workplace bullying or violence were related to an increased risk of depressive disorders (1). Another systematic review showed that high job demands, work-family conflict, and bullying or violence were related to psychotropic medication use (5). Additionally, several studies have related suicidal behaviors, including suicide deaths and attempts, to low job control (6, 7) and work-related sexual harassment (8), despite the majority of studies in this area focusing on suicidal ideation (9). Less attention has been paid to stressors that are specific for person-related work and their potential impact on mental health problems and suicidal behaviors of the exposed workers.

Person-related work is defined as occupations that require face-to-face or voice-to-voice interaction with individuals who are not employed at the workplace (ie, third parties), such as patients, customers, clients, passengers, or students (10). Several characteristics of person-related work may affect workers’ mental health and potentially constitute a risk factor for suicidal behaviors. First, *general contact with people* of a third party is challenging, because it is unpredictable and, in many cases, requires emotional labor insofar as workers have to manage their emotional expressions to fulfill the requirements of the interpersonal interactions. Emotional labor is taxing because displayed emotions often do not align with those genuinely felt (10). Second, in certain person-related work, such as human service occupations including healthcare, social services, and education, high *emotional demands* are common (11). While these professional roles can be deemed particularly meaningful, they can be burdensome due to the requirement of empathy and emotional engagement with people who are in difficult or distressing situations (12). Third, workers in these occupations frequently address needs and problems of customers, who may be upset or angry at times, resulting in *conflicts or quarrels* (13).

There have been studies that examined the association of person-related work and related aspects (such as emotional demands) with mental health problems, including depressive and anxiety disorders and the use of antidepressant medication (14–17). However, the majority of the studies used self-reported measures of exposure, which might have introduced bias and issues of reverse causation. An alternative way of assessing potential work stressors is using a job-exposure matrix (JEM), which evaluates the exposure based on the content of occupations. Indeed, it has been shown that content-related emotional demands are less affected by mental health issues compared to perceived emotional demands (18, 19). With regards to suicidality, only two cross-sectional studies have been conducted. Both found associations between high perceived emotional labor and demands at work and suicidal ideation (20, 21). To our knowledge, few prospective studies have yet focused on person-related work and suicidal behaviors.

This study aimed to investigate the prospective relationship between three dimensions of person-related work – general contact with people, emotional demands, and conflicts/quarrels – in relation to an array of mental health problems, including diagnoses of depressive disorder, anxiety/stress-related disorders, alcohol-/drug-use disorders, and pharmacotherapy for these disorders, as well as suicide deaths and suicide attempts. We hypothesized that a higher exposure to each of these three dimensions is associated with a higher risk of each of these mental health and suicide outcomes.

## Methods

### Study population

The study population was derived from the Swedish Work, Illness, and labor-market Participation (SWIP) cohort, which consists of all (around 5.8 million) individuals aged 16–64 years and registered in Sweden during the baseline year of 2005. The cohort was built using data from several Swedish administrative and medical registers, including the total population register (22), the longitudinal integrated database for health insurance and labor market studies (LISA) register (23), the national patient register (24, 25), the prescribed drug register (26), the cause of death register (27), as well as information from earlier population censuses. Linkages between registers were made by Statistics Sweden using unique personal identification numbers.

Notably, the patient registers started from 1973 onwards and the prescribed drug register started from 2005, so medical histories prior to these years are not available. Additionally, diagnoses of mental disorders in the patient register tend to be more severe cases, typically those requiring hospitalization or specialized treatment. Therefore, we included pharmacotherapy for these mental disorders to also identify less acute and severe cases that are treated in primary care.

For the present study, we included individuals who were aged 20–60 years and had information on the job that they held in 2006. This baseline year was decided because the prescribed drug register covers the whole population only since the end of 2005. This resulted in a final study population of 3 636 586 individuals.

Ethical approval was obtained by the Regional Ethics Review Board in Stockholm, the reference numbers are 2017/1224-31, 2018/1675-32, and 2022/02725-02.

### Exposure

Yearly occupational information is available in the LISA register administered by Statistics Sweden from 2005 onwards, recorded based on the Swedish ISCO-88 four-digit classification of occupations. We extracted individuals’ occupational codes in 2006.

We assessed three dimensions of person-related work using a JEM, constructed for around 350 occupations and separately for women and men, based on the Swedish Work Environment Surveys (1997–2013). The dimension *general contact with people* refers to the frequency of workers’ contact with a third party; the dimension *emotional demands* refers to the frequency of workers’ contact with a third party who is ill or has serious problems; and the dimension *conflicts/quarrels* refers to the frequency of workers’ involvement in a conflict or quarrel with a third party. Specific questions and answer options are shown in the supplementary material (XXX), table S1. Intra-class correlation coefficients (ICC) of general contact with people, emotional demands, and conflicts/quarrels were 0.30, 0.54, and 0.22 among men and 0.39, 0.44, and 0.12 among women. We have applied the JEM in previous studies focusing on cardiovascular disease (28) and type 2 diabetes (29).

We linked the JEM to the study population using their occupational codes and then categorized each dimension into three levels, based on the tertiles distribution in women and men, respectively. The ten most common occupations for each level of the three dimensions in women and men are listed in supplementary tables S2–4. For both women and men, the most common occupations in the high exposure category across the three dimensions were found in healthcare, education, the service industry, and social work; however, notable sex differences emerged in specific occupational titles.

### Outcome

For mental health problems, we identified diagnoses from the national patient register for in- and out-patient care visits (24, 25), including depressive disorder [International Statistical Classification of Diseases and Related Health Problems, 10^th^ version (ICD-10) codes: F32–F34, F38–F39, excluding F32.3, F33.3], anxiety/stress-related disorders (ICD-10 codes: F40–F48), and alcohol-/drug-use disorders (ICD-10 codes: F10–F16, F18–F19, excluding 4^th^ digit .0 and .9). We also identified pharmacotherapy (26), mainly, though not exclusively, used for these disorders from the prescribed drug register, including antidepressants (ATC: N06A), anxiolytics (ATC: N05B), hypnotics and sedatives (ATC: N05C), and medication used to treat alcohol and opioid dependence (ATC: N07BB, N07BC).

For suicidal behaviors, we identified suicide attempts and deaths (ICD-10 codes: X60–X84, Y10–Y34) from the national patient register, including in-patient and out-patient care visits (24, 25), and cause of death register (27), respectively.

### Covariates

Information on sex, birth year, and country of birth (defined as born in Sweden or not) was identified from the total population register (22). Information on education, civil status, and income from work was obtained from LISA at baseline (23). Highest attained education was grouped into (i) primary and lower secondary school or less (≤9 years), (ii) secondary (10–11 years), (iii) upper-secondary (12 years), (iv) ≤2 years of post-secondary/university (13–14 years), and (v) >3 years of post-secondary/university (≥15 years). Civil status was categorized as married, unmarried, divorced, and widowed.

Individuals were linked to their parents to capture early-life socioeconomic position (SEP). We used information from the population and housing censuses from 1960 (for those born 1941–1954), 1970 (for those born 1955–1964), 1980 (for those born 1965–1974) and 1990 (for those born 1975–1989). Early-life SEP was operationalized based on the father’s occupation, or, if the father’s information was missing, the mother’s occupation. It was categorized as non-manual employees at a higher level, non-manual employees at an intermediate level, assistant non-manual employees, skilled manual workers, non-skilled manual workers, farmers, and those with no parental occupation documented.

History of mental disorder diagnoses, medication dispensations for mental disorders, and suicide attempts were defined as the presence of any diagnoses or dispensations mentioned above at any time point up until the start of follow-up identified from the national patient register (diagnoses and suicide attempts) (24, 25) or prescribed drug register (medications) (26).

Job control and social support at work in 2006 were assessed using JEM based on the Swedish Work Environment Surveys (1997–2013) (30), and both were dichotomized using the median in the study population.

### Statistical analysis

We explored baseline characteristics of the study population according to the mental health and suicide outcomes by the end of the follow-up period, as well as the levels of dimensions of person-related work.

We treated each outcome as a separate end point and estimated the incidence rate of each outcome for each level of the three person-related work variables. We used Cox proportional hazard regression models with age as the underlying timescale to estimate hazard ratios (HR) and 95% confidence intervals (CI) for associations of the person-related work variables and the respective outcomes. Person-time was counted from 1 January 2007 until the first date when an outcome was recorded, death, emigration, age 65, or the end of the follow-up period on 31 December 2020, whichever came first.

It is known that men and women tend to hold different occupations and positions and may experience different exposures even within the same occupations (31). This is particularly relevant in the context of person-related work because many of the occupations are female-dominated. Furthermore, the lifelong risks of mental health problems and suicidal behaviors differ between the two sexes as well. Interaction terms between person-related work variables and sex were statistically significant for all outcomes (P<0.05). Therefore, all analyses were performed for men and women separately.

Model 1 was adjusted for birth year, birth country, education, civil status, early-life SEP, as well as job control because low job control has been found to be present in some person-related work and has been associated with mental health issues (11). Model 2 was additionally adjusted for income from work in 2006 and the history of mental health problems and/or suicide attempts specific to the outcome. That is, the history of depressive disorder, anxiety/stress-related disorders, alcohol-/drug-use disorders, and pharmacotherapy was respectively adjusted for in analyses looking at each of these outcomes. The history of any of the mental health problems and suicide attempts was adjusted for in analyses looking at suicide deaths and suicide attempts.

Person-related work encompassed both lower and higher qualified occupations, and occupational class has been associated with mental health outcomes (1, 32). Therefore, we explored the effect modification of occupational class in the association. We also explored effect modification by social support as in our previous studies (28, 29). Further, while we treated each dimension as an independent variable, considering their potential correlations, we explored whether the impact of exposure to a high level of one dimension remains in the absence of exposure to a high level of the other two dimensions. Furthermore, we conducted two sensitivity analyses. First, since we only considered occupational exposure at baseline, we excluded individuals who were aged <30 years because they were presumably more likely to change occupations. Second, we excluded anyone with a history of any mental health problems or suicide attempts to address potential reverse causation.

A P-value of <0.05 was used to determine statistical significance. To account for multiple testing, we applied Bonferroni correction to adjust the P-value by dividing 0.05 by 100. P<0.0005 was additionally used to confirm statistically significant associations.

Data management and statistical analyses were conducted using STATA 17 (StataCorp LLC, College Station, TX, USA.), and figures were created using R (version 4.3.1) package *ggplot2*.

## Results

From 2007 to 2020, 1 481 900 individuals (63.2% women) had at least one of the studied mental health outcomes, including 189 210 cases of depressive disorder, 276 653 cases of anxiety/stress-related disorders, 95 203 cases of alcohol-/drug-use disorders, 1 426 924 cases of pharmacotherapy for mental disorders, 7174 suicide deaths and 50 616 cases of suicide attempts. Among both women and men, those who had any of the outcomes, compared to those who did not, were more likely to have lower education, low job control, and a history of mental health problems or suicide attempts, less likely to be partnered, and had a lower income from work (table 1). A similar pattern was present when looking at specific mental health outcomes. In addition, women and men who had a diagnosis of depressive or anxiety/stress-related disorders, or suicide attempt during the follow-up period were younger than those who did not (supplementary tables S5–10).

**Table 1:** Baseline characteristics of the study population according to any mental health outcomes (including diagnoses of depressive disorder, anxiety/stress-related disorders, alcohol-/drug-use disorders, and pharmacotherapy for such disorders, as well as suicide deaths and suicide attempts) by the end of follow-up period. [SD=standard deviation.]

		Women						Men				
		No (N=952 233)			Yes (N=896 133)			No (N=1 202 453)			Yes (N=585 767)	
		%	Mean (SD)		%	Mean (SD)		%	Mean (SD)		%	Mean (SD)
Age (years)												
	20–29	18.5			17.4			17.8			16.8	
	30–39	25.8			26.1			27.6			26.5	
	40–49	25.6			28.9			26.2			29.6	
	50–60	30.1			27.6			38.4			27.1	
Foreign born		10.9			13.7			10.3			13.2	
Civil status												
	Married	46.3			42.9			41.9			39.1	
	Unmarried	42.9			41.6			49.6			49.7	
	Divorced	9.8			14.4			8.1			10.9	
	Widowed	1.0			1.1			0.4			0.3	
Education (years)												
	<10	8.7			10.8			13.7			16.6	
	10–11	25.4			28.3			28.2			30.5	
	12	22.9			22.3			24.0			22.3	
	13–14	17.4			16.2			14.9			13.5	
	≥15	25.4			22.2			18.9			16.8	
	Missing	0.2			0.2			0.3			0.3	
Parental socioeconomic positions												
	Higher non-manual	6.6			6.5			6.7			6.7	
	Intermediate non-manual	18.5			17.5			18.9			17.9	
	Assistant non-manual	10.6			10.6			10.7			10.7	
	Farmers	5.7			4.0			5.2			3.7	
	Skilled manual	23.4			22.9			23.5			22.8	
	Unskilled manual	22.7			22.9			22.9			22.8	
	No registered occupation	12.5			15.6			12.1			15.4	
Low job control		47.7			52.5			48.4			54.0	
History of depressive disorder		0.4			5.1			0.3			4.0	
History of anxiety/stress-related disorders		1.4			7.6			1.0			6.0	
History of alcohol-/drug-use disorders		0.3			2.1			0.8			4.6	
History of pharmacotherapy		3.1			32.8			2.0			24.5	
History of suicide attempts		0.9			3.6			0.9			2.9	
Income from work (in 100 Swedish krona) ^a^			2179.7 (1343.8)			1875.9 (1368.1)			3109.2 (2231.3)			2753.4 (2319.6)

^a^ Swedish krona = 0.11 euro in 2006.

Among both women and men, those with high exposure to general contact with people or conflicts/quarrels were higher educated. Those with high exposure to emotional demands were more likely to be foreign born and have low job control. Those with high exposure to emotional demands or conflicts/quarrels were more likely to have a history of mental health problems and suicide attempts. Finally, men with high exposure to the three dimensions and women with high exposure to conflicts/quarrels had a lower income from work (supplementary tables S11–13).

Overall, incidence rates were the highest for pharmacotherapy for mental disorders, followed by anxiety/stress-related disorders, depressive disorder, alcohol-/drug-use disorders, suicide attempts, and suicide deaths. Incidence rates for depressive disorder, anxiety/stress-related disorders, and pharmacotherapy were higher in women than in men, while the opposite was observed for alcohol-/drug-use disorders, suicide attempts, and suicide deaths (table 2).

**Table 2:** Incidence rate per 100 000 person-years by dimensions of person-related work and mental health outcomes among women and men.

		Number of people	Depressive disorder		Anxiety/stress- related disorders		Alcohol/drug use disorders		Pharmacotherapy		Suicide death		Suicide attempt
			Incidence rate		Incidence rate		Incidence rate		Incidence rate		Incidence rate		Incidence rate
**Women**													
General contact with people													
	Low	618 743	450.8		702.3		129.8		5176.5		7.7		95.7
	Medium	607 073	609.4		933.8		178.0		6005.5		11.1		126.1
	High	622 550	538.6		812.8		150.0		5684.0		10.5		110.3
Emotional demands													
	Low	608 851	459.6		728.5		131.7		4973.1		7.8		96.0
	Medium	624 641	508.8		807.1		132.3		5458.1		7.7		99.6
	High	614 874	630.6		911.8		194.0		6489.7		13.9		136.6
Conflicts/quarrels													
	Low	632 322	465.3		726.4		127.5		5167.4		8.0		96.0
	Medium	610 084	519.0		786.3		158.1		5611.8		10.3		112.1
	High	605 960	616.9		939.0		173.0		6110.2		11.1		124.4
**Men**													
General contact with people													
	Low	595 827	308.9		431.4		261.6		2915.3		22.5		121.0
	Medium	590 994	301.1		430.7		278.8		2962.1		21.1		110.6
	High	601 399	393.6		545.3		299.0		3519.0		23.3		117.4
Emotional demands													
	Low	591 501	263.1		373.6		235.4		2722.8		20.4		107.8
	Medium	611 785	311.0		447.6		275.5		2985.6		22.0		116.6
	High	584 934	434.5		592.2		330.2		3733.7		24.6		124.9
Conflicts/quarrels													
	Low	600 229	310.9		435.2		277.3		2935.5		23.2		121.5
	Medium	594 531	289.5		414.8		259.6		2931.8		20.5		109.4
	High	593 460	404.8		559.3		302.5		3538.9		23.4		118.1

**Figure 1:**
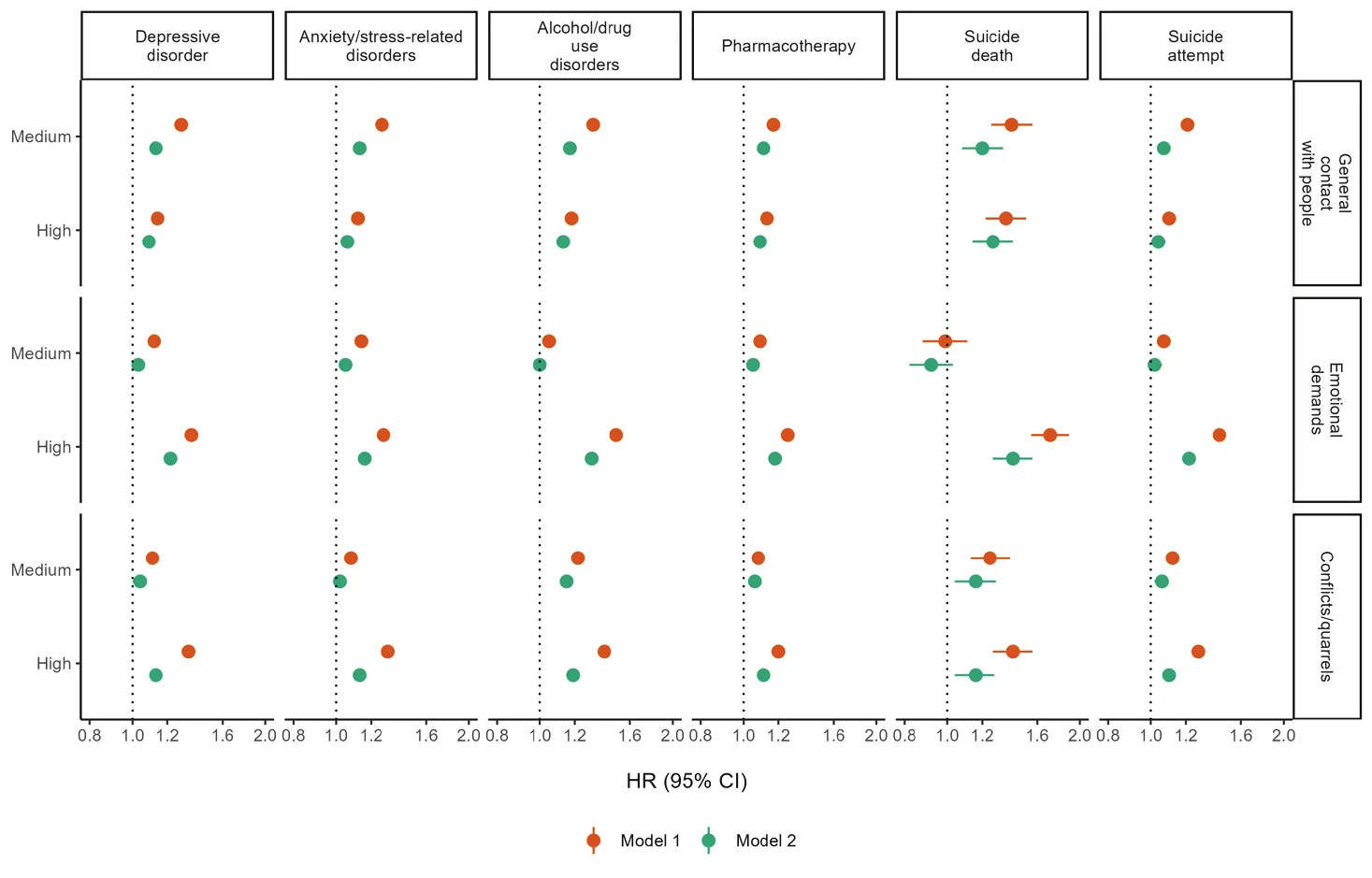
Hazard ratios (95% CI) for mental health outcomes by dimensions of person-related work among women. Model 1 adjusted for birth year, birth country, education, civil status, early-life socioeconomic position, and job control. Model 2 additionally adjusted for income from work and history of mental health problem and/or suicide attempts specific to the outcome. Reference groups include: low general contact with people, low emotional demands, and low conflicts/quarrels, respectively.

With regards to relative risk among women, in model 1, there was a relatively consistent dose–response pattern for the associations between emotional demands and conflicts/quarrels and all outcomes studied. This pattern was not observed for general contact with people – the risk for all outcomes was highest among those with a medium level of exposure, followed by those with a high and low level of exposure. After adjusting for income from work and history of mental health or suicide outcomes in model 2, the estimates were somewhat attenuated, but the observed patterns and associations remained statistically significant. A high exposure to general contact with people, emotional demands and conflicts/quarrels was respectively associated with 4–27%, 16–41%, and 11–19% increased risks of the studied mental health and suicide outcomes. However, the association between conflicts/quarrels and suicide deaths and the association between general contact with people and suicide attempts were less certain because their P-values were not <0.0005 (figure 1 and tables 3a & 3b).

**Table 3a:** Hazard ratios (HR) and 95% confidence intervals (CI) for depressive, anxiety/stress–related, and alcohol-/drug-use disorders by dimensions of person–related work among women.

		Depressive disorder				Anxiety/stress–related disorders				Alcohol-/drug-use disorders		
		Model 1 ^a^		Model 2 ^b^		Model 1 ^a^		Model 2 ^b^		Model 1 ^a^		Model 2 ^b^
		HR (95% CI)		HR (95% CI)		HR (95% CI)		HR (95% CI)		HR (95% CI)		HR (95% CI)
General contact with people												
	Low	Ref		Ref		Ref		Ref		Ref		Ref
	Medium	1.29 (1.27–1.31) ^†^		1.13 (1.11–1.14) ^†^		1.27 (1.25–1.28) ^†^		1.13 (1.12–1.15) ^†^		1.32 (1.29–1.36) ^†^		1.17 (1.14–1.20) ^†^
	High	1.14 (1.13–1.16) ^†^		1.09 (1.07–1.10) ^†^		1.12 (1.10–1.13) ^†^		1.06 (1.05–1.07) ^†^		1.18 (1.15–1.21) ^†^		1.13 (1.10–1.16) ^†^
Emotional demands												
	Low	Ref		Ref		Ref		Ref		Ref		Ref
	Medium	1.12 (1.10–1.13) ^†^		1.03 (1.01–1.04)		1.14 (1.12–1.15) ^†^		1.05 (1.04–1.07) ^†^		1.05 (1.02–1.08)		1.00 (0.97–1.02)
	High	1.36 (1.34–1.38) ^†^		1.22 (1.20–1.23) ^†^		1.28 (1.27–1.30) ^†^		1.16 (1.15–1.17) ^†^		1.49 (1.46–1.53) ^†^		1.31 (1.28–1.35) ^†^
Conflicts/quarrels												
	Low	Ref		Ref		Ref		Ref		Ref		Ref
	Medium	1.11 (1.09–1.13) ^†^		1.04 (1.00–1.05) ^†^		1.08 (1.07–1.10) ^†^		1.02 (1.01–1.03) ^†^		1.22 (1.18–1.25) ^†^		1.15 (1.12–1.18) ^†^
	High	1.34 (1.32–1.36) ^†^		1.13 (1.12–1.15) ^†^		1.31 (1.29–1.32) ^†^		1.13 (1.12–1.15) ^†^		1.40 (1.36–1.43) ^†^		1.19 (1.16–1.22) ^†^

^a^ Adjusted for birth year, birth country, education, civil status, early–life socioeconomic position, and job control. ^b^ Model 1 and additionally adjusted for income from work and history of mental health problem specific to the outcome. ^†^ P<0.0005.

**Table 3b:** Hazard ratios (HR) and 95% confidence intervals (CI) for pharmacotherapy, suicide death, and suicide attempt by dimensions of person–related work among women.

		Pharmacotherapy				Suicide death				Suicide attempt		
		Model 1 ^a^		Model 2 ^b^		Model 1 ^a^		Model 2 ^b^		Model 1 ^a^		Model 2 ^b^
		HR (95% CI)		HR (95% CI)		HR (95% CI)		HR (95% CI)		HR (95% CI)		HR (95% CI)
General contact with people												
	Low	Ref		Ref		Ref		Ref		Ref		Ref
	Medium	1.17 (1.16–1.18) ^†^		1.11 (1.10–1.11) ^†^		1.40 (1.26–1.56) ^†^		1.20 (1.08–1.34)		1.21 (1.17–1.25) ^†^		1.07 (1.04–1.10) ^†^
	High	1.13 (1.12–1.14) ^†^		1.09 (1.08–1.10) ^†^		1.36 (1.22–1.51) ^†^		1.27 (1.14–1.41) ^†^		1.10 (1.07–1.14) ^†^		1.04 (1.01–1.08)
Emotional demands												
	Low	Ref		Ref		Ref		Ref		Ref		Ref
	Medium	1.09 (1.08–1.10) ^†^		1.05 (1.04–1.06) ^†^		0.99 (0.88–1.11)		0.92 (0.82–1.03)		1.07 (1.04–1.11) ^†^		1.02 (0.99–1.06)
	High	1.26 (1.25–1.27) ^†^		1.18 (1.17–1.19) ^†^		1.71 (1.55–1.89) ^†^		1.41 (1.27–1.56) ^†^		1.43 (1.39–1.47) ^†^		1.22 (1.18–1.25) ^†^
Conflicts/quarrels												
	Low	Ref		Ref		Ref		Ref		Ref		Ref
	Medium	1.08 (1.07–1.09) ^†^		1.06 (1.05–1.07) ^†^		1.25 (1.13–1.39) ^†^		1.16 (1.04–1.29)		1.12 (1.09–1.16) ^†^		1.06 (1.02–1.09)
	High	1.20 (1.19–1.21) ^†^		1.11 (1.10–1.12) ^†^		1.41 (1.27–1.56) ^†^		1.16 (1.04–1.28)		1.28 (1.25–1.32) ^†^		1.10 (1.07–1.13) ^†^

^a^ Adjusted for birth year, birth country, education, civil status, early–life socioeconomic position, and job control. ^b^ Model 1 and additionally adjusted for income from work and history of mental health problem and/or suicide attempt specific to the outcome. ^†^ P<0.0005.

**Figure 2:**
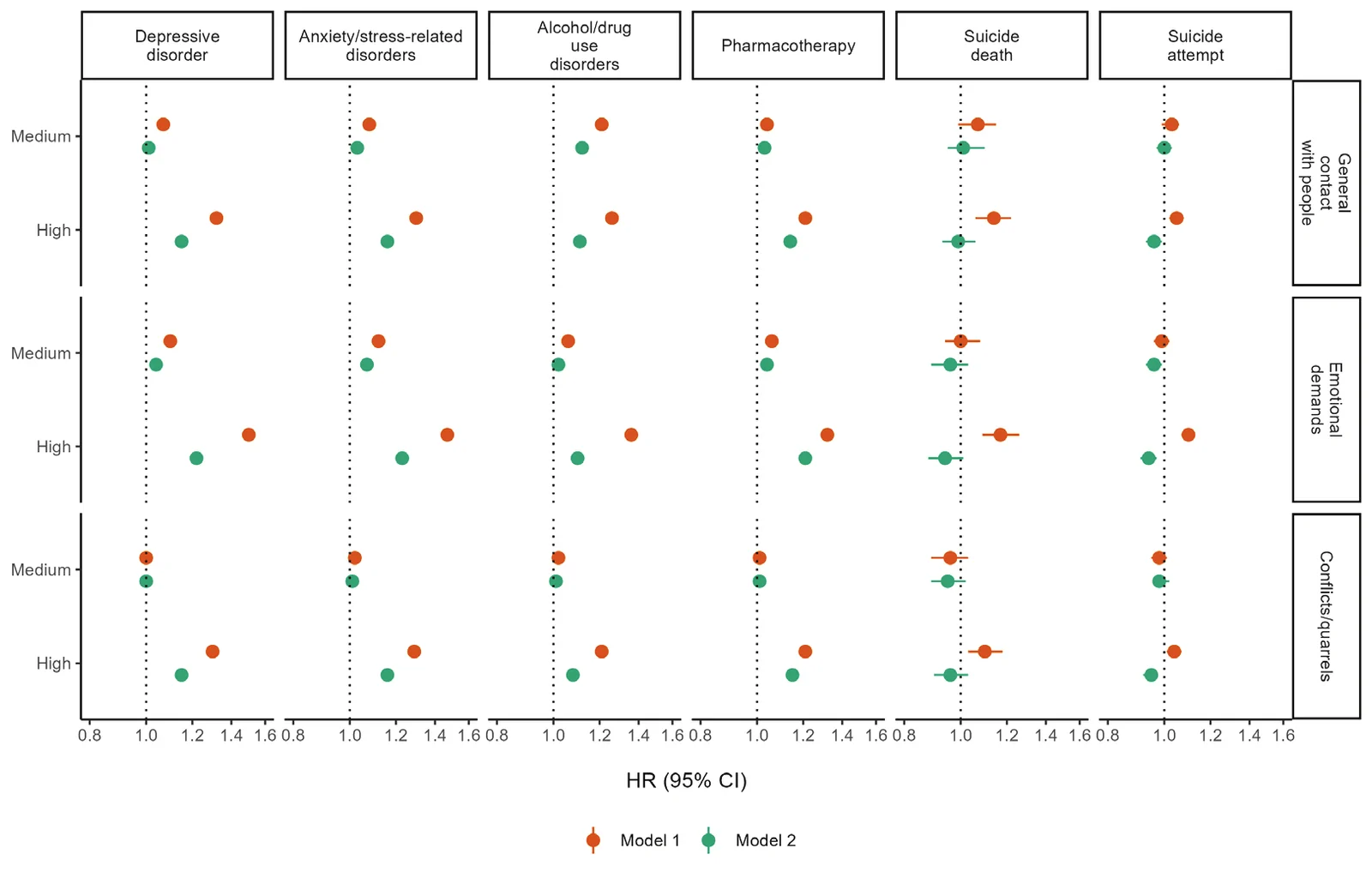
Hazard ratios (95% CIs) for mental health outcomes by dimensions of person-related work among men. Model 1 adjusted for birth year, birth country, education, civil status, early-life socioeconomic position, and job control. Model 2 additionally adjusted for income from work and history of mental health problem and/or suicide attempts specific to the outcome. Reference groups include: low general contact with people, low emotional demands, and low conflicts/quarrels, respectively.

Among men men, there was a clear dose–response association between the three dimensions of person-related work and mental health diagnoses and pharmacotherapy after adjusting for covariates. A high exposure to general contact with people, emotional demands and conflicts/quarrels was respectively associated with 11–16%, 10–23%, and 8–16% increased risks of these outcomes in model 2. Regarding suicide deaths and attempts, an increased risk was observed among those with high exposure to the three dimensions of person-related work in model 1; however, the increased risk disappeared after adjusting for either income from work or history of mental health problems and suicide attempts (figure 2 and tables 4a & 4b).

**Table 4a:** Hazard ratios (HR) and 95% confidence intervals (CI) for depressive, anxiety/stress–related, and alcohol-/drug-use disorders by dimensions of person–related work among men.

		Depressive disorder				Anxiety/stress–related disorders				Alcohol-/drug-use disorders		
		Model 1 ^a^		Model 2 ^b^		Model 1 ^a^		Model 2 ^b^		Model 1 ^a^		Model 2 ^b^
		HR (95% CI)		HR (95% CI)		HR (95% CI)		HR (95% CI)		HR (95% CI)		HR (95% CI)
General contact with people												
	Low	Ref		Ref		Ref		Ref		Ref		Ref
	Medium	1.07 (1.05–1.09) ^†^		1.01 (0.99–1.02)		1.08 (1.06–1.10) ^†^		1.03 (1.01–1.04)		1.21 (1.18–1.23) ^†^		1.12 (1.10–1.14) ^†^
	High	1.32 (1.29–1.34) ^†^		1.15 (1.13–1.17) ^†^		1.30 (1.28–1.32) ^†^		1.16 (1.14–1.18) ^†^		1.26 (1.23–1.28) ^†^		1.11 (1.09–1.13) ^†^
Emotional demands												
	Low	Ref		Ref		Ref		Ref		Ref		Ref
	Medium	1.10 (1.08–1.12) ^†^		1.04 (1.02–1.06) ^†^		1.12 (1.11–1.14) ^†^		1.07 (1.05–1.09) ^†^		1.06 (1.04–1.08) ^†^		1.02 (1.00–1.04)
	High	1.50 (1.47–1.53) ^†^		1.22 (1.20–1.24) ^†^		1.47 (1.45–1.50) ^†^		1.23 (1.22–1.25) ^†^		1.36 (1.33–1.39) ^†^		1.10 (1.08–1.13) ^†^
Conflicts/quarrels												
	Low	Ref		Ref		Ref		Ref		Ref		Ref
	Medium	1.00 (0.98–1.02)		1.00 (0.98–1.01)		1.02 (1.00–1.03)		1.01 (0.99–1.03)		1.02 (1.00–1.04)		1.01 (0.99–1.03)
	High	1.30 (1.28–1.33) ^†^		1.15 (1.13–1.17) ^†^		1.29 (1.27–1.31) ^†^		1.16 (1.14–1.18) ^†^		1.21 (1.19–1.23) ^†^		1.08 (1.06–1.10) ^†^

^a^ Adjusted for birth year, birth country, education, civil status, early–life socioeconomic position, and job control. ^b^ Model 1 and additionally adjusted for income from work and history of mental health problem specific to the outcome. ^†^ P<0.0005.

**Table 4b:** Hazard ratios (HR) and 95% confidence intervals (CI) for pharmacotherapy, suicide death, and suicide attempt by dimensions of person–related work among men.

		Pharmacotherapy				Suicide death				Suicide attempt		
		Model 1 ^a^		Model 2 ^b^		Model 1 ^a^		Model 2 ^b^		Model 1 ^a^		Model 2 ^b^
		HR (95% CI)		HR (95% CI)		HR (95% CI)		HR (95% CI)		HR (95% CI)		HR (95% CI)
General contact with people												
	Low	Ref		Ref		Ref		Ref		Ref		Ref
	Medium	1.04 (1.03–1.05) ^†^		1.03 (1.02–1.04) ^†^		1.07 (0.99–1.15)		1.01 (0.95–1.10)		1.03 (0.99–1.06)		1.00 (0.97–1.03)
	High	1.21 (1.20–1.22) ^†^		1.14 (1.13–1.15) ^†^		1.14 (1.06–1.22) ^†^		0.99 (0.93–1.06)		1.05 (1.02–1.08)		0.96 (0.93–0.99)
Emotional demands												
	Low	Ref		Ref		Ref		Ref		Ref		Ref
	Medium	1.06 (1.05–1.07) ^†^		1.04 (1.03–1.05) ^†^		1.00 (0.94–1.08)		0.96 (0.89–1.03)		0.99 (0.96–1.02)		0.96 (0.93–0.99)
	High	1.32 (1.31–1.33) ^†^		1.21 (1.20–1.22) ^†^		1.17 (1.09–1.26) ^†^		0.94 (0.88–1.01)		1.10 (1.07–1.13) ^†^		0.94 (0.91–0.97) ^†^
Conflicts/quarrels												
	Low	Ref		Ref		Ref		Ref		Ref		Ref
	Medium	1.01 (1.00–1.02)		1.01 (1.00–1.02) ^†^		0.96 (0.89–1.03)		0.95 (0.89–1.02)		0.98 (0.95–1.01)		0.98 (0.96–1.02)
	High	1.21 (1.20–1.22) ^†^		1.15 (1.14–1.16) ^†^		1.10 (1.03–1.18)		0.96 (0.90–1.03)		1.04 (1.01–1.07)		0.95 (0.92–0.97) ^†^

^a^ Adjusted for birth year, birth country, education, civil status, early–life socioeconomic position, and job control. ^b^ Model 1 and additionally adjusted for income from work and history of mental health problem and/or suicide attempt specific to the outcome. ^†^ P<0.0005.

The observed associations were generally present among blue- and white-collar workers, although the associations between conflicts/quarrels and mental health diagnoses appeared to be present only among blue-collar workers (supplementary tables S14–15). No effect modification by social support was observed (supplementary tables S16–17). The individual impact of high exposure to each dimension on mental health problems (including mental health diagnoses and pharmacotherapy) remained, although the estimate was small (supplementary table S18). The associations remained robust in the sensitivity analyses where individuals who were aged >30 years or had a history of mental health problems or suicide attempts were excluded (supplementary tables S19–20).

## Discussion

### Summary of results

In this register-based cohort study, we investigated the associations between three dimensions of person-related work and an array of mental health problems and suicidal behaviors. We found that, among both women and men, a high level of exposure to general contact with people, emotional demands, and conflicts/quarrels was respectively associated with a slightly increased risk of diagnoses of depressive disorder, anxiety/stress-related disorders, alcohol-/drug-use disorders, pharmacotherapy for these disorders, suicide deaths and suicide attempts. Estimates seemed to be strongest for emotional demands. When adjusting for income from work and history of mental health issues, the associations for suicide deaths and attempts remained among women but were no longer present among men.

### Interpretation and comparison with previous studies

While there have been studies investigating mental health problems in relation to dimensions of person-related work, the literature has mixed studies that are restricted to occupations with a high level of people-related work with studies that include all types of jobs. Some studies were conducted within specific occupations – such as healthcare (33), homecare (34) or call center workers (35) – and some studies assessed mental health symptoms through self-reports (13, 14, 36). Moreover, emotional demands might have a different meaning in different types of study populations. While most studies used a self-reported measure of the studied exposure (15, 16, 19), studies that employed a JEM have different definitions of exposures as well. For example, emotional demands in our JEM were based on work content (ie, the extent of contacting people with problems), and the JEM in another study concerned perceived burden with emotional demands (17).

Our findings that general contact with people was associated with adverse mental health problems may be explained by the emotional labor that is often required in person-related work. Interactions with a third party at work often involve implicit and explicit behavioral expectations regarding the appropriate expression or suppression of emotions, as dictated by societal, occupational, and organizational norms (37). Such emotional labor is taxing because there is a mismatch between the emotions that must be displayed and those genuinely felt (10). A French study found that a high level of self-reported emotional labor (ie, the need of hiding emotions at work) was associated with a higher risk of generalized anxiety disorders but not depressive disorder (15).

Working with individuals who are ill or facing serious personal challenges, which is common in human service occupations, can be particularly emotionally demanding. Professionals such as healthcare workers and social workers often bear responsibility for meeting clients’ fundamental needs and are regularly exposed to human suffering (38). Over time, this emotional burden may lead to compassion fatigue and burnout, particularly due to the lack of reciprocity in relationships with clients and patients (39). Among three Danish studies identified, one found that healthcare and educational workers, compared to workers in other types of occupations, had a higher risk of using antidepressants. The increased risk appeared to be explained by perceived emotional demands but not emotional labor (16). Another Danish prospective study showed that high self-reported perceived emotional demands, but not content-related emotional demands, were associated with a higher risk of depressive disorder (19). The other Danish study, similar to our study, utilized register data and a JEM and showed a positive association between emotional demands and the risk of depressive disorder (17). However, as mentioned previously, items used to create their JEM referred to perceived emotional demands, while the item used in our JEM referred to content-related emotional demands.

It has been shown that human service workers tend to encounter work-related threats and violence (40), and previous research has linked employment in specific human service roles, including healthcare, social work, and education, with an elevated risk of mental health problems and suicidal behaviors (16, 41–43). Our findings regarding the mental health impact of being involved in conflicts/quarrels support the notion that workers’ mental health may deteriorate in the context of conflict, quarrels, or aggression received from angry clients (13).

With regards to suicidality, two cross-sectional studies focused on several aspects of person-related work and suicidal ideation. A Korean study showed that workers who experienced higher emotional labor were more likely to experience suicidal ideation (20); a French study found that women, but not men, who reported higher emotional demands, comprising contact with people in general and with people in distress, emotional labor, and exposure to tensions and aggression, were more likely to report thoughts of death, suicide ideation, and suicide attempts (21). To our knowledge, the current study is the first to demonstrate the prospective association between person-related work and suicide deaths and attempts.

We found that a higher exposure to the three dimensions was associated with a higher risk of suicide deaths and attempts among women, while among men the association attenuated after adjusting for income from work or previous mental health problems and suicide attempts. These findings seem to be in line with the French study, where the association between high emotional demands and suicidal ideation was present among women but not men after adjusting for occupational class and depressive symptoms (21). It is known that there is sex segregation in the labor market, and, in our data, the occupations that were most represented in the different exposure levels of the studied dimensions of person-related work differed between women and men. Also, men who worked in occupations that are categorized as high exposure to the three dimensions had a lower income from work, while this was not the case among women. Thus, our findings may suggest that men with mental health issues are more selected into person-related work and/or that income plays a stronger role in men’s suicidal behavior in connection with person-related work.

However, the fact that women and men score high in the three studied dimensions of person-related work in the contexts of different occupations, yet still mostly showed very similar associations of their exposure levels with the mental health outcomes, suggests that the JEM assess the exposures as intended and the associations reflect the impact of these exposures rather than other characteristics of the occupations. Overall, our findings support the hypothesis that high levels of exposure to the three dimensions of person-related work contribute to an increased risk of mental health problems and suicidal behaviors among women and also an increased risk of mental health problems among men.

In our previous publications using the same data sources, we found that these dimensions of person-related work were associated with increased risks of cardiovascular disease (28) and type 2 diabetes (29). Taken together, these results strengthen the assumption of common mechanisms, such as a dysregulation of the hypothalamic–pituitary–adrenal stress axis and inflammatory processes (44, 45). In addition, these outcomes are inter-correlated (46), and thus adverse working conditions might cause one disease that subsequently leads to another. However, we are unable to rule out residual confounding underlying all these associations.

### Strengths and limitations

The current study benefits from several strengths. The use of a nationally representative sample helps minimize selection and attrition bias. The application of JEM to assess three dimensions of person-related work and job control enables more objective measurements, reducing the influence of other factors and reporting bias. Also, the items used concern content-related emotional demands, which, in our view, are more suitable for constructing a JEM than items concerning perceived emotional demands (18). Rather than targeting specific occupations, our approach categorizes occupations based on shared characteristics in person-related work exposures across three dimensions. We examined an array of mental health problems, pharmacotherapy for mental disorders, as well as suicidal behaviors, and our results showed a rather consistent association across the outcomes. Finally, the analysis accounts for key confounding factors, including early-life SEP and prior mental health or suicide history.

Some limitations of the study should be acknowledged. JEM assess exposures at the occupational level and therefore do not capture individual variations in work experiences or environments within a given occupation. Additionally, we were unable to measure workers’ actual emotional responses or reactivity when interacting with a third party. These limitations likely contribute to non-differential misclassification of workplace exposures, which may have led to an underestimation of the true associations and contributed to the modest associations observed in our study. For each dimension of person-related work, only a single item was used, which may not fully capture the complexity of each construct. Moreover, occupational exposures were measured at a single time point, without accounting for potential changes in occupation over time. However, our prior research has shown that psychosocial work environments at the occupational level tend to remain relatively stable over time (47). Additionally, in the current study, we found similar results when focusing on individuals aged ≥30 years who were presumably less likely to change jobs. This also means that adjusting for the prior occurrence of the outcome under study could constitute an over-adjustment, since many individuals already worked in the occupation for a longer period. Therefore, we also presented model 1. Additionally, we could not account for part-time work due to a lack of data. A lower income may be due to part-time work and the adjustment for income in the analyses may therefore be partly an adjustment for working hours. Finally, the dependence on the register data means that we could only include treated cases.

### Concluding remarks

In conclusion, person-related work is associated with a slightly increased risk of mental health problems and suicidal behaviors among women and with mental health problems among men. These findings underscore that the psychological demands and challenges inherent in person-related occupations pose a risk to workers’ mental health. Preventive strategies aimed at mitigating the mental health burden and suicide risk should be prioritized in occupations involving frequent human contact.

## Supplementary material

Supplementary material
