# Supplementary material for: Mental health problems and suicidal behaviors in person-related work: a Swedish register-based cohort study
Source: Scand J Work Environ Health. 2026 Aug 31;52(5):528–39. doi: 10.5271/sjweh.4316 (PMC13543172; doi:10.5271/sjweh.4316)
Supplement: Supplementary file 1 [file SJWEH-52-528-S001.pdf]

Mental health problems and suicidal behaviors in person-related work: a Swedish register-based cohort study[1]

by Kuan-Yu Pan, PhD,[2] Alicia Nevriana, PhD, Katrina Blindow, PhD, Melody Almroth, PhD, Katarina Kjellberg, PhD, Daniel Falkstedt, PhD

1. Supplementary Material
2. Correspondence to: Kuan-Yu Pan, PhD, Institute of Environmental Medicine, Karolinska Institutet, Solnavägen 4, 113 65 Stockholm, Sweden. [E-mail: kuan-yu.pan@ki.se].

Table S1. Questions and answer options in the Swedish Work Environment Surveys (1997-2013) by dimensions of person-related work

Dimension	Question	Answer options
General contact with people	Do you have anything to do with people at work who are not employed at the workplace (e.g., patients, customers, clients, passengers, students, etc.)?	Not at all, roughly 1/10 of the time, roughly ¼ of the time, half of the time, roughly ¾ of the time, almost all the time
Emotional demands	Does it happen that through work you come into close contact with the seriously ill or people with serious problems?	Not at all/rarely the last 3 months, a few days per month, one day per week, a few days per week, every day
Conflicts/quarrels	Are you involved in any form of conflict or quarrel in your workplace with other people (e.g., patients, customers, clients, passengers, students)?	Not at all the last 12 months, sometime the last 12 months, a few times the last 3 months, a few days per month, one day per week, a few days per week, every day

Table S2. Ten most common occupations according to sex and level of general contact with people at work

Sex	Exposure level	Swedish version of ISCO-88 Code	Occupational title	% in the total population
Women	Low	4190	Other office clerks	11.7
		9122	Helpers and cleaners in offices, hotels and other establishments	9.2
		4120	Numerical clerks	7.2
		4112	Office secretaries	4.9
		3431	Administrative secretaries and related associate professionals	4.6
		2470	Public service administrative professionals	4.4
		5122	Cooks	3.0
		2411	Accountants	2.5
		3433	Bookkeepers	2.4
		2310	College, university and higher education teaching professionals	2.3
	Medium	5133	Home-based personal care and related workers	24.5
		5131	Child-care workers	15.5
		3310	Pre-primary education teaching associate professionals	12.3
		5134	Attendants, psychiatric care	8.1
		9130	Helpers in restaurants	7.3
		3415	Technical and commercial sales representatives	3.4
		3418	Banking associate professionals	2.7
		2323	Teaching professionals, artistic and practical subjects	2.0
		3443	Government social benefits officials	1.9
		2340	Special education teaching professionals	1.8
	High	5132	Assistant nurses and hospital care and related workers	24.3
		2330	Primary education teaching professionals	10.8
		5222	Shop salespersons non-food stores	8.7
		3239	Nursing associate professionals not elsewhere classified	6.9
		5221	Shop salespersons, food stores	6.8
		2492	Social work professionals	3.4
		4222	Receptionists	2.7
		2221	Medical doctors	2.2
		4211	Cashiers and ticket clerks	2.0
		2321	Secondary education teaching professionals	1.9
Men	Low	2131	Computer systems designers, analysts and programmers	7.6
		4131	Store clerks and storekeepers	7.3
		8211	Machine tool operators	6.4
		3119	Physical and engineering science technicians not elsewhere classified	4.6
		8290	Other machine operators and assemblers	3.9
		7233	Agricultural- or industrial-machinery mechanics and fitters	3.4
		9320	Manufacturing laborers	3.2
		3115	Mechanical engineering technicians	2.9

		9190	Other sales and services elementary occupations	2.9
		4150	Mail carriers and sorting clerks	2.7
	Medium	7123	Carpenters and joiners	6.5
		3121	Computer assistants	4.5
		7231	Motor vehicle mechanics and fitters	4.3
		7136	Building and related electricians	3.5
		4190	Other office clerks	3.2
		2470	Public service administrative professionals	3.0
		2310	College, university and higher education teaching professionals	2.9
		1210	Directors and chief executives	2.9
		3112	Civil engineering technicians	2.7
		9122	Helpers and cleaners in offices, hotels and other establishments	2.5
	High	3415	Technical and commercial sales representatives	8.9
		8323	Heavy truck and lorry drivers	8.1
		5222	Shop salespersons non-food stores	5.7
		7137	Building caretakers	5.2
		5133	Home-based personal care and related workers	4.6
		2330	Primary education teaching professionals	3.6
		5221	Shop salespersons, food stores	3.2
		1314	Managers of small enterprises in wholesale and retail trade, hotels and restaurants, transport and communications	3.0
		5134	Attendants, psychiatric care	2.7
		2221	Medical doctors	2.6

Table S3. Ten most common occupations according to sex and level of emotional demands

Sex	Exposure level	Swedish version of ISCO-88 Code	Occupational title	% in the total population
Women	Low	4190	Other office clerks	11.9
		4120	Numerical clerks	7.3
		9130	Helpers in restaurants	7.3
		5221	Shop salespersons, food stores	7.0
		3415	Technical and commercial sales representatives	3.4
		3418	Banking associate professionals	2.7
		2411	Accountants	2.5
		3433	Bookkeepers	2.5
		2310	College, university and higher education teaching professionals	2.3
		2451	Authors, journalists and other writers	2.1
	Medium	5131	Child-care workers	15.0
		3310	Pre-primary education teaching associate professionals	11.9
		2330	Primary education teaching professionals	10.7
		9122	Helpers and cleaners in offices, hotels and other establishments	9.2
		5222	Shop salespersons non-food stores	8.6
		4112	Office secretaries	4.8
		3431	Administrative secretaries and related associate professionals	4.5
		2470	Public service administrative professionals	4.4
		5122	Cooks	2.9
		4222	Receptionists	2.7
	High	5132	Assistant nurses and hospital care and related workers	24.7
		5133	Home-based personal care and related workers	24.2
		5134	Attendants, psychiatric care	8.0
		3239	Nursing associate professionals not elsewhere classified	7.0
		2492	Social work professionals	3.5
		2221	Medical doctors	2.2
		3461	Social work associate professionals	1.9
		3443	Government social benefits officials	1.9
		2340	Special education teaching professionals	1.8
		5135	Dental nurses	1.7
Men	Low	2131	Computer systems designers, analysts and programmers	7.6
		7123	Carpenters and joiners	6.5
		8211	Machine tool operators	6.5
		3121	Computer assistants	4.5
		7231	Motor vehicle mechanics and fitters	4.3
		7136	Building and related electricians	3.5
		7233	Agricultural- or industrial-machinery mechanics and fitters	3.4
		3112	Civil engineering technicians	2.7
		3114	Electronics and telecommunications engineering technicians	2.6
		7212	Welders and flame cutters	2.6

	Medium	3415	Technical and commercial sales representatives	8.7
		8323	Heavy truck and lorry drivers	7.9
		4131	Store clerks and storekeepers	7.1
		3119	Physical and engineering science technicians not elsewhere classified	4.5
		8290	Other machine operators and assemblers	3.8
		9320	Manufacturing laborers	3.1
		1314	Managers of small enterprises in wholesale and retail trade, hotels and restaurants, transport and communications	3.0
		3115	Mechanical engineering technicians	2.9
		2310	College, university and higher education teaching professionals	2.8
		9190	Other sales and services elementary occupations	2.8
	High	5222	Shop salespersons non-food stores	5.9
		7137	Building caretakers	5.3
		5133	Home-based personal care and related workers	4.8
		2330	Primary education teaching professionals	3.7
		5221	Shop salespersons, food stores	3.2
		4190	Other office clerks	3.2
		2470	Public service administrative professionals	3.0
		1210	Directors and chief executives	2.9
		5134	Attendants, psychiatric care	2.8
		2221	Medical doctors	2.7

Table S4. Ten most common occupations according to sex and level of conflicts/quarrels

Sex	Exposure level	Swedish version of ISCO-88 Code	Occupational title	% in the total population
Women	Low	9122	Helpers and cleaners in offices, hotels and other establishments	9.0
		5222	Shop salespersons non-food stores	8.5
		4120	Numerical clerks	7.1
		4112	Office secretaries	4.8
		2470	Public service administrative professionals	4.3
		3415	Technical and commercial sales representatives	3.2
		5122	Cooks	2.9
		3418	Banking associate professionals	2.6
		2411	Accountants	2.4
		3433	Bookkeepers	2.4
	Medium	5132	Assistant nurses and hospital care and related workers	24.8
		4190	Other office clerks	11.8
		9130	Helpers in restaurants	7.3
		3239	Nursing associate professionals not elsewhere classified	7.1
		5221	Shop salespersons, food stores	6.9
		3431	Administrative secretaries and related associate professionals	4.6
		4222	Receptionists	2.8
		4211	Cashiers and ticket clerks	2.1
		2321	Secondary education teaching professionals	2.0
		2412	Personnel and careers professionals	1.9
	High	5133	Home-based personal care and related workers	24.6
		5131	Child-care workers	15.5
		3310	Pre-primary education teaching associate professionals	12.3
		2330	Primary education teaching professionals	11.1
		5134	Attendants, psychiatric care	8.1
		2492	Social work professionals	3.5
		2323	Teaching professionals, artistic and practical subjects	2.0
		3461	Social work associate professionals	1.9
		3443	Government social benefits officials	1.9
		4223	Telephone switchboard operators	1.8
Men	Low	2131	Computer systems designers, analysts and programmers	7.5
		4131	Store clerks and storekeepers	7.2
		7123	Carpenters and joiners	6.4
		8211	Machine tool operators	6.4
		7136	Building and related electricians	3.4
		9190	Other sales and services elementary occupations	2.9
		4150	Mail carriers and sorting clerks	2.7
		3114	Electronics and telecommunications engineering technicians	2.6
		7212	Welders and flame cutters	2.6
		7241	Electrical mechanics fitters and services	2.3
	Medium	3415	Technical and commercial sales representatives	9.0

		8323	Heavy truck and lorry drivers	8.1
		7137	Building caretakers	5.2
		3121	Computer assistants	4.4
		7231	Motor vehicle mechanics and fitters	4.3
		8290	Other machine operators and assemblers	3.9
		7233	Agricultural- or industrial-machinery mechanics and fitters	3.4
		2470	Public service administrative professionals	3.0
		3115	Mechanical engineering technicians	2.9
		2310	College, university and higher education teaching professionals	2.9
	High	5222	Shop salespersons non-food stores	5.8
		5133	Home-based personal care and related workers	4.7
		3119	Physical and engineering science technicians not elsewhere classified	4.7
		2330	Primary education teaching professionals	3.6
		9320	Manufacturing laborers	3.2
		5221	Shop salespersons, food stores	3.2
		4190	Other office clerks	3.2
		1314	Managers of small enterprises in wholesale and retail trade, hotels and restaurants, transport and communications	3.1
		5134	Attendants, psychiatric care	2.8
		2221	Medical doctors	2.7

Table S5. Baseline characteristics of the study population according to depression diagnosis by the end of follow-up period

	Women				Men			
	No	Yes	No	Yes	No	Yes	No	Yes
	1,731,289	117,077	1,731,289	117,077	1,716,087	72,133	1,716,087	72,133
Age	%	%	Mean (SD)	Mean (SD)	%	%	Mean (SD)	Mean (SD)
20-29	17.5	25.0			17.3	22.2		
30-39	25.7	29.3			27.2	28.6		
40-49	27.2	27.3			27.2	29.1		
50-60	29.6	18.4			28.3	20.1		
Foreign born	12.0	16.0			11.1	14.9		
Civil status								
Partnered	45.4	33.6			41.4	31.3		
Unmarried	41.7	49.9			49.4	56.8		
Divorced	11.8	15.7			8.9	11.6		
Widowed	1.1	0.8			0.3	0.3		
Education (years)								
<10	9.6	12.6			14.5	18.1		
10-11	26.8	26.7			28.9	30.6		
12	22.4	25.0			23.4	23.9		
13-14	16.9	15.7			14.5	13.1		
≥15	24.1	19.8			18.4	14.0		
Missing	0.2	0.2			0.3	0.3		
Parental socioeconomic positions								
Higher non-manual	6.5	6.8			6.7	6.7		
Intermediate non-manual	18.1	17.6			18.6	17.4		
Assistant non-manual	10.6	10.3			10.8	10.3		
Farmers	5.0	2.8			4.7	2.9		
Skilled manual	23.3	21.7			23.3	22.1		

Unskilled manual	22.8	22.4			22.9	22.9		
No registered occupation	13.7	18.4			13.0	17.7		
Low job control	49.4	59.4			49.7	62.2		
History of depressive disorder	1.5	19.8			0.9	16.3		
History of anxiety/stress-related disorders	3.4	19.4			2.1	14.9		
History of alcohol/drug use disorders	0.9	5.8			1.7	10.1		
History of pharmacotherapy	15.1	53.2			8.0	41.9		
History of suicide attempts	1.8	8.8			1.4	6.2		
Income from work (in 100 Swedish krona)			2073.7 (1363.8)	1422.4 (1214.9)			3029.3 (2277.3)	2120.8 (1791.0)

1 Swedish krona = 0.11 Euro in 2006

Table S6. Baseline characteristics of the study population according to anxiety/stress-related diagnosis by the end of follow-up period

	Women				Men			
	No	Yes	No	Yes	No	Yes	No	Yes
	1,671,996	176,370	1,671,996	176,370	1,687,937	100,283	1,687,937	100,283
Age	%	%	Mean (SD)	Mean (SD)	%	%	Mean (SD)	Mean (SD)
20-29	17.2	25.7			17.1	24.1		
30-39	25.4	31.2			27.0	31.1		
40-49	27.1	27.6			27.2	28.8		
50-60	30.3	15.5			28.7	16.0		
Foreign born	11.9	15.5			11.0	14.9		
Civil status								
Partnered	45.7	34.3			41.5	31.2		
Unmarried	41.4	50.8			49.1	58.4		
Divorced	11.8	14.2			9.0	10.2		
Widowed	1.1	0.7			0.4	0.2		
Education (years)								
<10	9.6	12.2			14.4	17.8		
10-11	26.9	25.7			28.9	30.0		
12	22.3	25.7			23.4	25.0		
13-14	16.9	15.8			14.5	12.9		
≥15	24.1	20.3			18.5	14.0		
Missing	0.2	0.3			0.3	0.3		
Parental socioeconomic positions								
Higher non-manual	6.5	6.9			6.7	6.6		
Intermediate non-manual	18.1	18.0			18.6	17.8		
Assistant non-manual	10.6	10.6			10.8	10.6		
Farmers	5.0	2.5			4.8	2.5		
Skilled manual	23.3	21.9			23.3	22.2		

Unskilled manual	22.8	22.3			22.9	22.8		
No registered occupation	13.7	17.8			12.9	17.5		
Low job control	49.2	57.8			49.6	61.2		
History of depressive disorder	1.7	12.1			1.0	9.7		
History of anxiety/stress-related disorders	3.0	17.7			1.9	15.1		
History of alcohol/drug use disorders	0.8	4.8			1.6	8.5		
History of pharmacotherapy	14.9	42.8			7.8	34.7		
History of suicide attempts	1.7	6.9			1.4	5.1		
Income from work (in 100 Swedish krona)			2086.8 (1363.9)	1517.3 (1253.5)			3037.2 (2280.8)	2242.6 (1862.4)

1 Swedish krona = 0.11 Euro in 2006

Table S7. Baseline characteristics of the study population according to alcohol/drug use diagnosis by the end of follow-up period

	Women				Men			
	No	Yes	No	Yes	No	Yes	No	Yes
	1,813,796	34,570	1,813,796	34,570	1,727,587	60,633	1,727,587	60,633
Age	%	%	Mean (SD)	Mean (SD)	%	%	Mean (SD)	Mean (SD)
20-29	18.0	19.1			17.4	19.0		
30-39	26.0	25.9			27.4	24.3		
40-49	27.1	31.9			27.2	31.3		
50-60	28.9	23.1			28.0	25.4		
Foreign born	12.2	12.2			11.2	11.2		
Civil status								
Partnered	45.0	29.7			41.5	25.7		
Unmarried	42.2	48.0			49.4	57.6		
Divorced	11.8	21.0			8.8	16.3		
Widowed	1.0	1.3			0.3	0.4		
Education (years)								
<10	9.6	17.8			14.3	24.5		
10-11	26.6	35.0			28.7	36.1		
12	22.6	21.6			23.5	20.5		
13-14	16.9	12.3			14.6	9.6		
≥15	24.1	13.0			18.6	8.9		
Missing	0.2	0.3			0.3	0.4		
Parental socioeconomic positions								
Higher non-manual	6.5	5.7			6.7	5.4		
Intermediate non-manual	18.1	16.4			18.6	16.4		
Assistant non-manual	10.6	10.7			10.7	10.9		
Farmers	4.9	2.0			4.8	2.2		
Skilled manual	23.1	24.3			23.2	25.0		

Unskilled manual	22.8	25.6			22.8	25.8		
No registered occupation	14.0	15.3			13.2	14.3		
Low job control	49.7	63.5			49.7	63.9		
History of depressive disorder	2.4	17.2			1.3	9.1		
History of anxiety/stress-related disorders	4.1	23.2			2.3	12.6		
History of alcohol/drug use disorders	0.8	22.4			1.2	24.6		
History of pharmacotherapy	16.8	55.0			8.4	35.4		
History of suicide attempts	1.9	16.4			1.4	8.3		
Income from work (in 100 Swedish krona)			2043.6 (1363.5)	1447.1 (1265.8)			3021.7 (2274.6)	2164.4 (1845.9)

1 Swedish krona = 0.11 Euro in 2006

Table S8. Baseline characteristics of the study population according to pharmacotherapy by the end of follow-up period

	Women				Men			
	No	Yes	No	Yes	No	Yes	No	Yes
	976,274	872,092	976,274	872,092	1,233,388	554,832	1,233,388	554,832
Age	%	%	Mean (SD)	Mean (SD)	%	%	Mean (SD)	Mean (SD)
20-29	18.7	17.1			17.9	16.4		
30-39	25.9	26.1			27.6	26.5		
40-49	25.6	29.0			26.2	29.7		
50-60	29.8	27.8			28.3	27.4		
Foreign born	10.9	13.7			10.3	13.2		
Civil status								
Partnered	46.1	43.1			41.6	39.5		
Unmarried	43.1	41.3			49.9	49.2		
Divorced	9.8	14.5			8.2	11.0		
Widowed	1.0	1.1			0.3	0.3		
Education (years)								
<10	8.8	10.9			13.8	16.5		
10-11	25.3	28.4			28.2	30.5		
12	22.9	22.2			24.1	22.1		
13-14	17.4	16.2			14.9	13.6		
≥15	25.4	22.1			18.7	17.0		
Missing	0.2	0.2			0.3	0.3		
Parental socioeconomic positions								
Higher non-manual	6.6	6.4			6.7	6.8		
Intermediate non-manual	18.6	17.4			18.8	17.9		
Assistant non-manual	10.6	10.5			10.8	10.7		
Farmers	5.6	4.0			5.2	3.6		
Skilled manual	23.3	23.0			23.5	22.7		

Unskilled manual	22.7	23.0			22.9	22.8		
No registered occupation	12.6	15.7			12.1	15.5		
Low job control	47.8	52.5			48.7	53.7		
History of depressive disorder	0.5	5.2			0.4	4.1		
History of anxiety/stress-related disorders	1.5	7.7			1.1	6.1		
History of alcohol/drug use disorders	0.4	2.1			0.9	4.5		
History of pharmacotherapy	3.2	33.6			2.0	25.6		
History of suicide attempts	1.0	3.6			1.0	2.8		
Income from work (in 100 Swedish krona)			2172.7 (1343.7)	1875.4 (1369.7)			3096.6 (2227.0)	2761.5 (2336.2)

1 Swedish krona = 0.11 Euro in 2006

Table S9. Baseline characteristics of the study population according to suicide death by the end of follow-up period

	Women				Men			
	No	Yes	No	Yes	No	Yes	No	Yes
	1,846,128	2,238	1,846,128	2,238	1,783,284	4,936	1,783,284	4,936
Age	%	%	Mean (SD)	Mean (SD)	%	%	Mean (SD)	Mean (SD)
20-29	18.0	15.8			17.5	17.3		
30-39	26.0	25.6			27.3	26.0		
40-49	27.2	36.6			27.3	33.8		
50-60	28.8	22.0			28.0	22.9		
Foreign born	12.2	13.1			11.2	9.8		
Civil status								
Partnered	44.7	31.1			41.0	28.7		
Unmarried	42.3	47.1			49.6	57.8		
Divorced	12.0	20.4			9.0	13.1		
Widowed	1.0	1.4			0.4	0.4		
Education (years)								
<10	9.7	13.9			14.6	20.8		
10-11	26.8	32.7			28.9	35.8		
12	22.6	20.6			23.5	20.6		
13-14	16.8	15.2			14.5	11.3		
≥15	23.9	17.4			18.2	11.3		
Missing	0.2	0.2			0.3	0.2		
Parental socioeconomic positions								
Higher non-manual	6.5	6.5			6.7	5.5		
Intermediate non-manual	18.0	15.7			18.5	16.8		
Assistant non-manual	10.6	9.7			10.7	9.7		
Farmers	4.8	3.5			4.7	4.7		
Skilled manual	23.2	24.1			23.3	25.6		

Unskilled manual	22.8	25.5			22.9	25.1		
No registered occupation	14.1	15.0			13.2	12.6		
Low job control	50.0	60.4			50.2	62.9		
History of depressive disorder	2.7	25.0			1.5	13.2		
History of anxiety/stress-related disorders	4.4	28.3			2.6	13.9		
History of alcohol/drug use disorders	1.2	20.2			2.0	15.7		
History of pharmacotherapy	17.5	58.8			9.3	34.9		
History of suicide attempts	2.2	24.6			1.6	11.0		
Income from work (in 100 Swedish krona)			2033.2 (1364.0)	1402.1 (1312.7)			2994.6 (2267.8)	2274.5 (1694.5)

1 Swedish krona = 0.11 Euro in 2006

Table S10. Baseline characteristics of the study population according to suicide attempt by the end of follow-up period

	Women				Men			
	No	Yes	No	Yes	No	Yes	No	Yes
	1,823,229	25,137	1,823,229	25,137	1,762,741	25,479	1,762,741	25,479
Age	%	%	Mean (SD)	Mean (SD)	%	%	Mean (SD)	Mean (SD)
20-29	17.9	24.5			17.3	26.1		
30-39	25.9	27.9			27.3	28.5		
40-49	27.2	28.6			27.3	27.5		
50-60	29.0	19.0			28.1	17.9		
Foreign born	12.2	13.6			11.2	10.9		
Civil status								
Partnered	44.8	34.4			41.1	30.0		
Unmarried	42.2	48.0			49.5	58.8		
Divorced	12.0	16.8			9.0	10.9		
Widowed	1.0	0.8			0.4	0.3		
Education (years)								
<10	9.7	15.5			14.5	20.8		
10-11	26.7	31.2			28.9	33.5		
12	22.6	24.6			23.4	25.7		
13-14	16.9	13.5			14.5	10.0		
≥15	23.9	14.9			18.4	9.7		
Missing	0.2	0.3			0.3	0.3		
Parental socioeconomic positions								
Higher non-manual	6.5	5.5			6.7	4.9		
Intermediate non-manual	18.1	15.7			18.6	15.6		
Assistant non-manual	10.6	9.6			10.7	10.2		
Farmers	4.9	3.3			4.7	4.3		
Skilled manual	23.1	24.5			23.3	24.9		

Unskilled manual	22.8	25.1			22.8	25.8		
No registered occupation	14.0	16.3			13.2	14.3		
Low job control	49.8	63.4			50.0	64.1		
History of depressive disorder	2.5	16.9			1.4	8.5		
History of anxiety/stress-related disorders	4.2	20.9			2.5	11.0		
History of alcohol/drug use disorders	1.0	12.5			1.9	13.6		
History of pharmacotherapy	17.2	44.6			9.1	25.2		
History of suicide attempts	2.0	19.0			1.4	12.8		
Income from work (in 100 Swedish krona)			2039.9 (1364.3)	1490.3 (1237.8)			3002.3 (2270.3)	2322.5 (1892.1)

1 Swedish krona = 0.11 Euro in 2006

Table S11. Baseline characteristics of the study population according to the level of general contact with people

	Women			Men		
	Low	Medium	High	Low	Medium	High
	618,743	607,073	622,550	595,827	590,994	601,399
Age	%	%	%	%	%	%
20-29	13.2	20.4	20.3	17.3	16.1	19.0
30-39	27.3	24.1	26.5	28.7	27.1	26.0
40-49	29.1	26.6	25.9	28.0	27.6	26.4
50-60	30.4	28.9	27.3	26.0	29.2	28.6
Foreign born	13.2	12.3	11.2	11.7	10.1	11.7
Civil status						
Partnered	46.3	43.7	44.0	38.9	42.7	41.4
Unmarried	40.3	43.0	43.5	52.6	48.0	48.4
Divorced	12.2	12.2	11.6	8.2	9.0	9.9
Widowed	1.2	1.1	0.9	0.3	0.3	0.3
Education (years)						
<10	12.9	10.3	6.1	17.3	13.7	12.9
10-11	26.5	29.0	24.9	31.9	29.9	25.0
12	22.6	22.7	22.5	22.8	23.7	23.8
13-14	14.9	20.7	15.0	13.0	13.4	17.0
≥15	22.8	17.1	31.4	14.7	18.9	21.1
Missing	0.3	0.2	0.1	0.3	0.4	0.2
Parental socioeconomic positions						
Higher non-manual	7.1	5.7	6.7	5.1	6.9	8.1
Intermediate non-manual	19.1	16.9	18.1	16.9	19.3	19.4
Assistant non-manual	10.5	10.3	10.9	8.9	10.7	12.6
Farmers	4.6	4.9	5.0	6.2	4.3	3.5
Skilled manual	22.4	23.8	23.3	24.6	24.7	20.6

Unskilled manual	21.5	24.0	23.0	24.6	21.9	22.2
No registered occupation	14.8	14.4	13.0	13.7	12.2	13.6
Low job control	63.6	48.2	38.2	62.4	34.3	53.8
History of depressive disorder	2.2	3.1	2.7	1.4	1.3	1.8
History of anxiety/stress-related disorders	3.7	5.1	4.4	2.6	2.3	3.1
History of alcohol/drug use disorders	0.9	1.5	1.2	2.0	1.9	2.2
History of pharmacotherapy	17.4	18.6	17.6	8.7	8.8	10.5
History of suicide attempts	1.8	2.5	2.3	1.7	1.4	1.7
	Mean (SD)	Mean (SD)	Mean (SD)	Mean (SD)	Mean (SD)	Mean (SD)
Income from work (in 100 Swedish krona)	2313.0 (1492.2)	1823.2 (1364.7)	1957.6 (1170.2)	2941.7 (1665.0)	3215.2 (2852.5)	2824.4 (2110.9)

1 Swedish krona = 0.11 Euro in 2006

Table S12. Baseline characteristics of the study population according to the level of emotional demands

	Women			Men		
	Low	Medium	High	Low	Medium	High
	608,851	624,641	614,874	591,501	611,785	584,934
Age	%	%	%	%	%	%
20-29	19.3	17.7	17.0	14.6	17.7	20.1
30-39	28.2	26.1	23.6	29.9	27.4	24.5
40-49	26.9	26.7	28.0	28.7	27.6	25.5
50-60	25.6	29.5	31.4	26.8	27.3	29.9
Foreign born	11.7	11.8	13.1	8.4	10.1	15.2
Civil status						
Partnered	42.4	46.2	45.4	42.1	40.1	40.7
Unmarried	45.7	41.4	39.7	49.5	50.6	48.7
Divorced	10.9	11.4	13.8	8.1	8.9	10.2
Widowed	1.0	1.0	1.1	0.3	0.4	0.4
Education (years)						
<10	12.7	9.8	6.8	14.1	16.9	12.8
10-11	26.6	23.3	30.5	31.3	31.9	23.4
12	27.1	20.8	19.9	22.4	24.8	23.1
13-14	14.0	19.4	17.0	13.9	12.6	16.9
≥15	19.4	26.5	25.6	18.0	13.5	23.4
Missing	0.2	0.2	0.2	0.3	0.3	0.4
Parental socioeconomic positions						
Higher non-manual	7.2	6.7	5.6	6.2	5.8	8.1
Intermediate non-manual	19.4	18.9	15.9	19.1	17.6	19.0
Assistant non-manual	11.0	11.3	9.4	9.9	11.0	11.3
Farmers	4.5	4.6	5.4	6.1	4.5	3.4
Skilled manual	22.6	22.8	24.0	25.4	24.1	20.2

Unskilled manual	21.9	22.2	24.4	22.8	24.8	21.0
No registered occupation	13.4	13.5	15.3	10.5	12.2	17.0
Low job control	39.2	51.7	58.9	35.7	52.5	62.6
History of depressive disorder	2.2	2.5	3.3	1.2	1.4	2.0
History of anxiety/stress-related disorders	3.7	4.2	5.5	2.1	2.5	3.3
History of alcohol/drug use disorders	1.0	1.0	1.6	1.6	2.0	2.5
History of pharmacotherapy	15.3	16.8	20.4	8.0	8.8	11.3
History of suicide attempts	1.8	1.9	2.9	1.4	1.6	1.8
	Mean (SD)	Mean (SD)	Mean (SD)	Mean (SD)	Mean (SD)	Mean (SD)
Income from work (in 100 Swedish krona)	2214.3 (1495.6)	1932.8 (1335.8)	1953.5 (1231.7)	3322.2 (2219.6)	2949.4 (1889.2)	2704.7 (2607.4)

1 Swedish krona = 0.11 Euro in 2006

Table S13. Baseline characteristics of the study population according to the level of conflicts/quarrels

	Women			Men		
	Low	Medium	High	Low	Medium	High
	632,322	610,084	605,960	600,229	594,531	593,460
Age	%	%	%	%	%	%
20-29	16.2	18.2	19.7	17.5	14.5	20.4
30-39	28.2	24.8	24.7	28.4	27.5	25.8
40-49	28.0	27.4	26.2	27.7	28.9	25.3
50-60	27.6	29.6	29.4	26.4	29.1	28.5
Foreign born	13.7	11.3	11.6	10.5	9.6	13.6
Civil status						
Partnered	45.2	44.6	44.2	38.8	43.4	40.7
Unmarried	42.2	42.2	42.4	52.4	47.0	49.5
Divorced	11.6	12.1	12.4	8.4	9.2	9.5
Widowed	1.0	1.1	1.0	0.4	0.4	0.3
Education (years)						
<10	12.8	8.0	8.3	16.4	16.1	11.4
10-11	23.5	31.4	25.5	33.6	30.3	22.8
12	23.3	25.5	19.0	23.9	22.4	23.9
13-14	14.9	15.0	20.7	12.1	12.8	18.6
≥15	25.2	20.0	26.3	13.7	18.0	23.0
Missing	0.3	0.1	0.2	0.3	0.4	0.3
Parental socioeconomic positions						
Higher non-manual	7.9	5.8	5.7	5.3	6.4	8.4
Intermediate non-manual	19.3	17.3	17.5	17.0	18.3	20.4
Assistant non-manual	10.6	10.6	10.5	9.3	10.8	12.1
Farmers	4.6	5.1	4.8	5.0	5.9	3.2
Skilled manual	21.7	24.0	23.9	26.2	23.1	20.4

Unskilled manual	20.7	24.0	23.9	24.6	23.8	20.2
No registered occupation	15.2	13.2	13.7	13.6	11.7	15.3
Low job control	46.4	54.8	48.9	56.7	36.6	57.4
History of depressive disorder	2.2	2.7	3.2	1.4	1.3	1.8
History of anxiety/stress-related disorders	3.7	4.4	5.2	2.5	2.4	3.0
History of alcohol/drug use disorders	0.9	1.2	1.5	2.1	1.8	2.2
History of pharmacotherapy	16.2	17.6	18.9	8.8	8.7	10.6
History of suicide attempts	1.8	2.2	2.6	1.7	1.5	1.6
	Mean (SD)	Mean (SD)	Mean (SD)	Mean (SD)	Mean (SD)	Mean (SD)
Income from work (in 100 Swedish krona)	2294.1 (1541.3)	1988.5 (1334.2)	1803.6 (1131.0)	2915.2 (1634.3)	3272.2 (2835.4)	2790.9 (2145.2)

1 Swedish krona = 0.11 Euro in 2006

Table S14. Hazard ratios (95% CIs) for mental health outcomes by dimensions of person-related work and occupational class in women

	**Depressive disorder**		**Anxiety/stress-related disorders**		**Alcohol/drug use disorders**	
General contact with people	White collar	Blue collar	White collar	Blue collar	White collar	Blue collar
Low	Ref	Ref	Ref	Ref	Ref	Ref
Medium	1.01 (0.98-1.03)	1.18 (1.16-1.21)†	1.02 (1.01-1.04)	1.23 (1.21-1.25)†	0.98 (0.94-1.03)	1.23 (1.19-1.28)†
High	1.13 (1.10-1.15)†	1.07 (1.05-1.10)†	1.05 (1.03-1.07)†	1.11 (1.09-1.13)†	1.12 (1.07-1.17)†	1.17 (1.12-1.21)†
Emotional demands						
Low	Ref	Ref	Ref	Ref	Ref	Ref
Medium	1.03 (1.01-1.06)	1.02 (1.00-1.05)	1.03 (1.01-1.05)	1.08 (1.06-1.09)†	0.99 (0.95-1.04)	0.98 (0.95-1.02)
High	1.25 (1.22-1.28)†	1.21 (1.18-1.23)†	1.10 (1.08-1.12)†	1.21 (1.20-1.23)†	1.25 (1.19-1.31)†	1.30 (1.26-1.35)†
Conflicts/quarrels						
Low	Ref	Ref	Ref	Ref	Ref	Ref
Medium	1.00 (0.98-1.02)	1.07 (1.05-1.09)†	0.99 (0.97-1.01)	1.06 (1.04-1.07)†	1.08 (1.04-1.13)†	1.19 (1.15-1.23)†
High	1.00 (0.97-1.01)	1.22 (1.19-1.24)†	1.00 (0.98-1.02)	1.23 (1.21-1.25)†	0.97 (0.93-1.02)	1.26 (1.22-1.30)†
	**Pharmacotherapy**		**Suicide death**		**Suicide attempt**	
General contact with people	White collar	Blue collar	White collar	Blue collar	White collar	Blue collar
Low	Ref	Ref	Ref	Ref	Ref	Ref
Medium	1.02 (1.01-1.03)†	1.16 (1.15-1.17)†	0.97 (0.81-1.16)	1.21 (1.04-1.40)	1.07 (1.01-1.13)	1.01 (0.97-1.05)
High	1.10 (1.09-1.11)†	1.09 (1.08-1.10)†	1.40 (1.19-1.65)†	1.16 (1.00-1.35)	1.14 (1.08-1.20)†	0.97 (0.93-1.01)
Emotional demands						
Low	Ref	Ref	Ref	Ref	Ref	Ref
Medium	1.06 (1.05-1.07)†	1.03 (1.02-1.04)†	0.90 (0.76-1.08)	0.90 (0.77-1.05)	1.08 (1.02-1.13)	0.97 (0.93-1.01)
High	1.17 (1.16-1.18)†	1.17 (1.16-1.18)†	1.45 (1.22-1.73)†	1.30 (1.13-1.48)†	1.25 (1.19-1.33)†	1.16 (1.12-1.21)†
Conflicts/quarrels						
Low	Ref	Ref	Ref	Ref	Ref	Ref
Medium	1.03 (1.02-1.04)†	1.07 (1.06-1.08)†	1.15 (0.98-1.35)	1.14 (0.99-1.32)	1.06 (1.01-1.12)	1.05 (1.01-1.09)
High	1.02 (1.01-1.03)†	1.16 (1.15-1.17)†	0.88 (0.74-1.05)	1.19 (1.04-1.36)	1.04 (0.98-1.09)	1.09 (1.05-1.13)†

Model 2 adjusted for birth year, birth country, education, civil status, early-life SEP, job control, income from work, and history of mental health problem and/or suicide attempts specific to the outcome

†p-value<0.0005

Table S15. Hazard ratios (95% CIs) for mental health outcomes by dimensions of person-related work and occupational class in men

	**Depressive disorder**		**Anxiety/stress-related disorders**		**Alcohol/drug use disorders**	
General contact with people	White collar	Blue collar	White collar	Blue collar	White collar	Blue collar
Low	Ref	Ref	Ref	Ref	Ref	Ref
Medium	1.12 (1.08-1.15)†	0.96 (0.94-0.99)	1.13 (1.10-1.16)†	0.99 (0.97-1.01)	1.14 (1.09-1.18)†	1.13 (1.10-1.16)†
High	1.14 (1.10-1.18)†	1.16 (1.13-1.19)†	1.15 (1.12-1.18)†	1.18 (1.16-1.21)†	1.18 (1.13-1.23)†	1.09 (1.07-1.12)†
Emotional demands						
Low	Ref	Ref	Ref	Ref	Ref	Ref
Medium	0.93 (0.90-0.96)†	1.11 (1.08-1.13)†	0.98 (0.95-1.01)	1.12 (1.09-1.14)†	1.02 (0.99-1.06)	1.02 (0.99-1.04)
High	1.16 (1.12-1.19)†	1.28 (1.25-1.31)†	1.14 (1.11-1.17)†	1.30 (1.27-1.33)†	1.10 (1.06-1.15)†	1.12 (1.10-1.15)†
Conflicts/quarrels						
Low	Ref	Ref	Ref	Ref	Ref	Ref
Medium	0.97 (0.94-1.01)	0.99 (0.96-1.01)	1.03 (1.00-1.06)	0.98 (0.97-1.00)	1.04 (1.00-1.08)	0.99 (0.96-1.01)
High	1.04 (1.01-1.07)	1.21 (1.18-1.23)†	1.04 (1.02-1.07)	1.23 (1.20-1.25)†	1.02 (0.98-1.06)	1.11 (1.09-1.14)†
	**Pharmacotherapy**		**Suicide death**		**Suicide attempt**	
General contact with people	White collar	Blue collar	White collar	Blue collar	White collar	Blue collar
Low	Ref	Ref	Ref	Ref	Ref	Ref
Medium	1.13 (1.11-1.14)†	0.98 (0.97-0.99)†	1.02 (0.88-1.17)	1.05 (0.97-1.14)	1.04 (0.98-1.11)	1.00 (0.97-1.04)
High	1.22 (1.21-1.24)†	1.11 (1.10-1.12)†	1.08 (0.95-1.24)	1.00 (0.92-1.08)	1.11 (1.04-1.18)	0.95 (0.92-0.99)
Emotional demands						
Low	Ref	Ref	Ref	Ref	Ref	Ref
Medium	1.03 (1.02-1.04)†	1.06 (1.05-1.07)†	1.06 (0.93-1.21)	0.94 (0.86-1.02)	1.08 (1.02-1.15)	0.93 (0.90-0.97)†
High	1.24 (1.23-1.25)†	1.20 (1.19-1.21)†	1.17 (1.03-1.33)	0.92 (0.84-1.00)	1.17 (1.10-1.24)†	0.92 (0.89-0.96)†
Conflicts/quarrels						
Low	Ref	Ref	Ref	Ref	Ref	Ref
Medium	1.03 (1.02-1.04)†	0.99 (0.98-1.00)	0.92 (0.80-1.06)	0.98 (0.90-1.06)	1.04 (0.98-1.11)	0.98 (0.95-1.02)
High	1.14 (1.12-1.15)†	1.16 (1.15-1.17)†	1.05 (0.92-1.19)	0.99 (0.91-1.08)	1.08 (1.02-1.15)	0.97 (0.93-1.00)

Model 2 adjusted for birth year, birth country, education, civil status, early-life SEP, job control, income from work, and history of mental health problem and/or suicide attempts specific to the outcome

†p-value<0.0005

Table S16. Hazard ratios (95% CIs) for mental health outcomes by dimensions of person-related work and social support in women

	**Depressive disorder**		**Anxiety/stress-related disorders**		**Alcohol/drug use disorders**	
General contact with people	High social support	Low social support	High social support	Low social support	High social support	Low social support
Low	Ref	Ref	Ref	Ref	Ref	Ref
Medium	1.00 (0.98-1.03)	1.19 (1.17-1.21)†	1.06 (1.03-1.08)†	1.17 (1.15-1.19)†	0.98 (0.94-1.02)	1.23 (1.19-1.28)†
High	1.05 (1.03-1.08)†	1.11 (1.09-1.14)†	1.05 (1.03-1.06)†	1.07 (1.05-1.09)†	1.09 (1.05-1.14)†	1.17 (1.12-1.23)†
Emotional demands						
Low	Ref	Ref	Ref	Ref	Ref	Ref
Medium	1.06 (1.04-1.08)†	1.00 (0.98-1.03)	1.10 (1.09-1.15)†	1.00 (0.98-1.02)	0.98 (0.95-1.02)	0.98 (0.93-1.03)
High	1.15 (1.13-1.18)†	1.25 (1.22-1.28)†	1.11 (1.09-1.30)†	1.17 (1.15-1.19)†	1.22 (1.18-1.27)†	1.30 (1.24-1.36)†
Conflicts/quarrels						
Low	Ref	Ref	Ref	Ref	Ref	Ref
Medium	1.05 (1.03-1.07)†	1.04 (1.01-1.06)	1.03 (1.01-1.04)†	1.01 (0.99-1.03)	1.12 (1.09-1.16)†	1.22 (1.16-1.28)†
High	1.08 (1.05-1.11)†	1.13 (1.11-1.15)†	1.11 (1.09-1.14)†	1.13 (1.11-1.14)†	0.98 (0.94-1.03)	1.21 (1.17-1.26)†
	**Pharmacotherapy**		**Suicide death**		**Suicide attempt**	
General contact with people	High social support	Low social support	High social support	Low social support	High social support	Low social support
Low	Ref	Ref	Ref	Ref	Ref	Ref
Medium	1.01 (1.00-1.02)	1.16 (1.15-1.17)†	0.99 (0.84-1.18)	1.21 (1.04-1.40)	0.95 (0.91-1.00)	1.08 (1.04-1.13)†
High	1.05 (1.04-1.06)†	1.12 (1.11-1.13)†	1.24 (1.07-1.44)	1.26 (1.06-1.49)	1.03 (0.99-1.08)	1.05 (1.00-1.10)
Emotional demands						
Low	Ref	Ref	Ref	Ref	Ref	Ref
Medium	1.07 (1.06-1.08)†	1.03 (1.02-1.04)†	0.85 (0.73-0.99)	0.98 (0.81-1.19)	0.96 (0.91-1.00)	1.03 (0.98-1.09)
High	1.13 (1.12-1.14)†	1.21 (1.20-1.22)†	1.23 (1.06-1.42)	1.48 (1.24-1.78)†	1.20 (1.14-1.25)†	1.17 (1.11-1.23)†
Conflicts/quarrels						
Low	Ref	Ref	Ref	Ref	Ref	Ref
Medium	1.04 (1.03-1.05)†	1.05 (1.04-1.06)†	1.19 (1.04-1.37)	1.12 (0.94-1.34)	1.09 (1.04-1.13)†	1.06 (1.00-1.12)
High	1.06 (1.05-1.07)†	1.11 (1.10-1.12)†	0.89 (0.73-1.09)	1.10 (0.96-1.26)	0.99 (0.94-1.05)	1.07 (1.02-1.11)

Model 2 adjusted for birth year, birth country, education, civil status, early-life SEP, job control, income from work, and history of mental health problem and/or suicide attempts specific to the outcome
†p-value<0.0005

Table S17. Hazard ratios (95% CIs) for mental health outcomes by dimensions of person-related work and social support in men

	**Depressive disorder**		**Anxiety/stress-related disorders**		**Alcohol/drug use disorders**	
General contact with people	High social support	Low social support	High social support	Low social support	High social support	Low social support
Low	Ref	Ref	Ref	Ref	Ref	Ref
Medium	1.01 (0.98-1.04)	1.10 (1.07-1.14)†	1.04 (1.01-1.06)	1.13 (1.10-1.16)†	1.11 (1.07-1.14)†	1.18 (1.14-1.22)†
High	1.17 (1.14-1.20)†	1.21 (1.17-1.25)†	1.21 (1.19-1.23)†	1.20 (1.17-1.23)†	1.18 (1.14-1.21)†	1.09 (1.05-1.12)†
Emotional demands						
Low	Ref	Ref	Ref	Ref	Ref	Ref
Medium	1.03 (1.01-1.07)	1.06 (1.03-1.09)†	1.07 (1.05-1.10)†	1.07 (1.04-1.09)†	1.07 (1.04-1.10)†	0.98 (0.95-1.01)
High	1.19 (1.16-1.22)†	1.30 (1.26-1.34)†	1.22 (1.19-1.25)†	1.29 (1.26-1.32)†	1.17 (1.14-1.21)†	1.07 (1.04-1.10)†
Conflicts/quarrels						
Low	Ref	Ref	Ref	Ref	Ref	Ref
Medium	0.99 (0.96-1.02)	1.02 (0.99-1.05)	1.03 (1.01-1.06)	1.02 (0.99-1.05)	1.02 (0.99-1.05)	1.00 (0.97-1.02)
High	1.15 (1.12-1.18)†	1.18 (1.15-1.21)†	1.18 (1.16-1.20)†	1.18 (1.15-1.21)†	1.13 (1.10-1.16)†	1.02 (0.98-1.05)
	**Pharmacotherapy**		**Suicide death**		**Suicide attempt**	
General contact with people	High social support	Low social support	High social support	Low social support	High social support	Low social support
Low	Ref	Ref	Ref	Ref	Ref	Ref
Medium	1.03 (1.02-1.04)†	1.07 (1.06-1.08)†	0.91 (0.81-1.02)	1.15 (1.03-1.29)	0.95 (0.91-1.00)	1.00 (0.96-1.05)
High	1.17 (1.16-1.18)†	1.15 (1.14-1.17)†	1.04 (0.95-1.13)	1.03 (0.91-1.16)	1.00 (0.96-1.04)	0.93 (0.89-0.98)
Emotional demands						
Low	Ref	Ref	Ref	Ref	Ref	Ref
Medium	1.07 (1.06-1.08)†	1.02 (1.01-1.03)†	0.98 (0.88-1.08)	0.95 (0.86-1.05)	1.03 (0.99-1.08)	0.90 (0.86-0.94)†
High	1.23 (1.21-1.23)†	1.22 (1.21-1.23)†	1.02 (0.92-1.12)	0.92 (0.83-1.01)	1.04 (1.00-1.09)	0.90 (0.86-0.94)†
Conflicts/quarrels						
Low	Ref	Ref	Ref	Ref	Ref	Ref
Medium	0.99 (0.98-1.00)	1.03 (1.02-1.05)†	0.94 (0.84-1.04)	0.97 (0.88-1.07)	1.00 (0.96-1.05)	0.95 (0.91-0.99)
High	1.17 (1.16-1.18)†	1.14 (1.13-1.15)†	0.99 (0.91-1.08)	0.97 (0.87-1.08)	1.03 (0.99-1.07)	0.87 (0.83-0.91)†

Model 2 adjusted for birth year, birth country, education, civil status, early-life SEP, job control, income from work, and history of mental health problem and/or suicide attempts specific to the outcome
†p-value<0.0005

Table S18. Hazard ratios (95% CIs) for mental health problems (including mental health diagnoses and pharmacotherapy) in men and women by dimensions of person-related work in the absence of high level of the other two dimensions

General contact with people	Men	Women
Low	Ref	Ref
Medium	1.00 (0.99-1.00)	1.01 (1.01-1.02)
High	1.01 (1.00-1.02)	1.01 (1.01-1.02)
Emotional demands		
Low	Ref	Ref
Medium	1.04 (1.03-1.05)†	1.04 (1.03-1.05)†
High	1.14 (1.12-1.15)†	1.03 (1.01-1.05)
Conflicts/quarrels		
Low	Ref	Ref
Medium	1.01 (1.00-1.02)	1.05 (1.04-1.06)†
High	1.06 (1.05-1.08)†	1.04 (1.03-1.05)†

The association for general contact with people is estimated among those without high level of exposure to emotional demands or conflicts/quarrels; The association for emotional demands is estimated among those without high level of exposure to general contact with people or conflicts/quarrels; The association for conflicts/quarrels is estimated among those without high level of exposure to general contact with people or emotional demands

Model 2 adjusted for birth year, birth country, education, civil status, early-life SEP, job control, income from work, and history of mental health problems

†p-value<0.0005

Table S19. Hazard ratios (95% CIs) for mental health outcomes by dimensions of person-related work in men and women aged 30 years or older at baseline

	**Depressive disorder**		**Anxiety/stress-related disorders**		**Alcohol/drug use disorders**	
General contact with people	Men	Women	Men	Women	Men	Women
Low	Ref	Ref	Ref	Ref	Ref	Ref
Medium	1.04 (1.02-1.07)†	1.11 (1.09-1.13)†	1.07 (1.05-1.09)†	1.12 (1.10-1.13)†	1.14 (1.12-1.17)†	1.12 (1.09-1.15)†
High	1.21 (1.19-1.24)†	1.10 (1.08-1.11)†	1.23 (1.21-1.25)†	1.07 (1.06-1.09)†	1.17 (1.14-1.19)†	1.14 (1.11-1.18)†
Emotional demands						
Low	Ref	Ref	Ref	Ref	Ref	Ref
Medium	1.05 (1.03-1.08)†	1.04 (1.02-1.06)†	1.08 (1.06-1.10)†	1.06 (1.04-1.07)†	1.02 (1.00-1.05)	0.99 (0.96-1.02)
High	1.29 (1.27-1.32)†	1.24 (1.21-1.26)†	1.31 (1.28-1.33)†	1.15 (1.14-1.17)†	1.15 (1.12-1.17)†	1.27 (1.23-1.31)†
Conflicts/quarrels						
Low	Ref	Ref	Ref	Ref	Ref	Ref
Medium	1.01 (0.99-1.03)	1.02 (1.01-1.04)	1.03 (1.01-1.05)	1.02 (1.00-1.03)	1.02 (1.00-1.04)	1.13 (1.10-1.17)†
High	1.21 (1.19-1.24)†	1.11 (1.09-1.13)†	1.24 (1.22-1.26)†	1.12 (1.10-1.13)†	1.12 (1.09-1.14)†	1.14 (1.11-1.18)†
	**Pharmacotherapy**		**Suicide death**		**Suicide attempt**	
General contact with people	Men	Women	Men	Women	Men	Women
Low	Ref	Ref	Ref	Ref	Ref	Ref
Medium	1.04 (1.04-1.05)†	1.08 (1.07-1.09)†	1.06 (0.98-1.15)	1.15 (1.02-1.29)	1.01 (0.97-1.05)	1.06 (1.02-1.10)
High	1.16 (1.16-1.17)†	1.09 (1.08-1.10)†	1.04 (0.96-1.12)	1.28 (1.14-1.44)†	1.01 (0.97-1.04)	1.07 (1.03-1.11)†
Emotional demands						
Low	Ref	Ref	Ref	Ref	Ref	Ref
Medium	1.04 (1.03-1.05)†	1.05 (1.04-1.06)†	0.94 (0.87-1.01)	0.91 (0.80-1.03)	0.97 (0.93-1.00)	1.03 (0.99-1.07)
High	1.23 (1.22-1.24)†	1.17 (1.16-1.18)†	0.98 (0.91-1.06)	1.38 (1.23-1.55)†	1.00 (0.97-1.04)	1.22 (1.18-1.27)†
Conflicts/quarrels						
Low	Ref	Ref	Ref	Ref	Ref	Ref
Medium	1.02 (1.01-1.03)†	1.04 (1.03-1.05)†	0.96 (0.89-1.03)	1.11 (0.99-1.25)	1.01 (0.97-1.04)	1.05 (1.01-1.09)
High	1.16 (1.15-1.17)†	1.09 (1.08-1.10)†	1.02 (0.94-1.09)	1.07 (0.96-1.20)	1.00 (0.97-1.04)	1.07 (1.04-1.11)†

Model 2 adjusted for birth year, birth country, education, civil status, early-life SEP, job control, income from work, and history of mental health problem and/or suicide attempts specific to the outcome

†p-value<0.0005

Table S20. Hazard ratios (95% CIs) for mental health outcomes by dimensions of person-related work in men and women, excluding anyone with a history of any mental health problems or suicide attempts

	Depressive disorder		Anxiety/stress-related disorders		Alcohol/drug use disorders	
General contact with people	Men	Women	Men	Women	Men	Women
Low	Ref	Ref	Ref	Ref	Ref	Ref
Medium	1.02 (1.00-1.05)	1.13 (1.11-1.16)†	1.05 (1.03-1.07)†	1.14 (1.12-1.16)†	1.17 (1.14-1.21)†	1.13 (1.08-1.18)†
High	1.18 (1.15-1.20)†	1.05 (1.03-1.08)†	1.20 (1.18-1.23)†	1.05 (1.03-1.07)†	1.14 (1.11-1.17)†	1.06 (1.01-1.11)
Emotional demands						
Low	Ref	Ref	Ref	Ref	Ref	Ref
Medium	1.03 (1.01-1.06)	1.04 (1.01-1.06)	1.08 (1.06-1.11)†	1.07 (1.05-1.09)†	1.03 (1.00-1.06)	0.99 (0.95-1.04)
High	1.23 (1.20-1.26)†	1.17 (1.14-1.20)†	1.27 (1.24-1.29)†	1.12 (1.10-1.14)†	1.16 (1.12-1.19)†	1.22 (1.17-1.27)†
Conflicts/quarrels						
Low	Ref	Ref	Ref	Ref	Ref	Ref
Medium	1.01 (0.98-1.03)	1.02 (1.00-1.04)	1.02 (1.00-1.05)	1.01 (0.99-1.03)	1.04 (1.01-1.07)	1.09 (1.05-1.14)†
High	1.16 (1.13-1.19)†	1.14 (1.12-1.17)†	1.19 (1.16-1.21)†	1.14 (1.12-1.16)†	1.11 (1.08-1.14)†	1.15 (1.10-1.20)†
	**Pharmacotherapy**		**Suicide death**		**Suicide attempt**	
General contact with people	Men	Women	Men	Women	Men	Women
Low	Ref	Ref	Ref	Ref	Ref	Ref
Medium	1.04 (1.03-1.05)†	1.12 (1.11-1.13)†	1.04 (0.94-1.14)	1.07 (0.89-1.29)	1.03 (0.99-1.07)	1.05 (1.01-1.10)
High	1.17 (1.16-1.18)†	1.09 (1.09-1.10)†	1.01 (0.92-1.11)	1.12 (0.94-1.34)	0.99 (0.95-1.03)	1.03 (1.00-1.08)
Emotional demands						
Low	Ref	Ref	Ref	Ref	Ref	Ref
Medium	1.05 (1.04-1.06)†	1.06 (1.05-1.07)†	1.03 (0.94-1.13)	0.78 (0.64-0.94)	0.97 (0.93-1.00)	1.03 (0.98-1.08)
High	1.22 (1.21-1.23)†	1.18 (1.17-1.19)†	0.94 (0.85-1.04)	1.22 (1.03-1.45)	0.97 (0.94-1.01)	1.22 (1.17-1.28)†
Conflicts/quarrels						
Low	Ref	Ref	Ref	Ref	Ref	Ref
Medium	1.02 (1.01-1.03)†	1.05 (1.04-1.06)†	0.99 (0.90-1.08)	1.09 (0.91-1.30)	0.99 (0.95-1.03)	1.06 (1.02-1.11)
High	1.16 (1.15-1.17)†	1.13 (1.12-1.14)†	0.95 (0.86-1.04)	1.08 (0.90-1.30)	0.97 (0.93-1.01)	1.10 (1.05-1.15)†

Model 2 adjusted for birth year, birth country, education, civil status, early-life SEP, job control, and income from work
†p-value<0.0005
